# Impact of the serrated pathway on the simulated comparative effectiveness of colorectal cancer screening tests

**DOI:** 10.1093/jncics/pkae077

**Published:** 2024-09-06

**Authors:** Reinier G S Meester, Uri Ladabaum

**Affiliations:** Division of Gastroenterology and Hepatology, Stanford University School of Medicine, Stanford, CA, USA; Health Economics and Outcomes Research, Freenome Holdings, Inc, South San Francisco, CA, USA; Division of Gastroenterology and Hepatology, Stanford University School of Medicine, Stanford, CA, USA

## Abstract

**Background:**

Colorectal cancers (CRCs) arise from adenomas, which can produce fecal occult blood and can be detected endoscopically, or sessile serrated lesions (SSLs), which rarely bleed and may be more challenging to detect. Models informing CRC screening policy should reflect both pathways, accounting for uncertainty.

**Methods:**

Novel decision-analytic model of the adenoma and serrated pathways for CRC (ANSER) to compare current and emerging screening strategies, accounting for differential test sensitivities for adenomas and SSLs, and uncertainty. Strategies included colonoscopy every 10 years, stool-DNA/FIT (sDNA-FIT) every 1-3 years, or fecal immunochemical testing (FIT) every year from age 45 to 75 years. Outcomes included CRC cases and deaths, cost-effectiveness (cost/quality-adjusted life-year [QALY] gained), and burden–benefit (colonoscopies/life-year gained), with 95% uncertainty intervals (UIs).

**Results:**

ANSER predicted 62.5 (95% UI = 58.8-66.3) lifetime CRC cases and 24.1 (95% UI = 22.5-25.7) CRC deaths/1000 45-year-olds without screening, and 78%-87% CRC mortality reductions with screening. The tests’ outcome distributions overlapped for QALYs gained but separated for required colonoscopies and costs. All strategies cost less than $100 000/QALY gained vs no screening. Colonoscopy was the most effective and cost-effective, costing $9300/life-year gained (95% UI = $500-$21 900) vs FIT. sDNA-FIT cost more than $500 000/QALY gained vs FIT. As more CRCs arose from SSLs, colonoscopy remained preferred based on clinical benefit and cost-effectiveness, but cost-effectiveness improved for a next-generation sDNA-FIT.

**Conclusion:**

When the serrated pathway is considered, modeling suggests that colonoscopy is cost-effective vs FIT. In contrast, modeling suggests that sDNA-FIT is not cost-effective vs FIT despite its greater sensitivity for SSLs, even if a substantial minority of CRCs arise from SSLs.

Colorectal cancer (CRC) remains the second leading cause of cancer death in the United States despite the proven benefit and acceptance of CRC screening (1). Most CRCs develop from adenomas (2), and CRC screening has relied primarily on CRC and adenoma detection through fecal occult blood or endoscopic visualization (3-7). It is now understood that a substantial minority of CRCs arise from sessile serrated lesions (SSLs), which rarely bleed, and which have a subtle endoscopic appearance (8,9).

The US Preventive Services Task Force (USPSTF) issues a Grade A recommendation for CRC screening, without preferential ranking of screening strategies (10). The alternatives, which include colonoscopy, fecal immunochemical testing (FIT), or stool-DNA/FIT (sDNA-FIT), differ in their sensitivity and specificity for CRC, adenomas, and SSLs (11), and they are recommended at different intervals. There are no published randomized clinical trials examining the comparative effectiveness of these CRC screening tests. Decision-analytic modeling integrates data on disease epidemiology and natural history, test performance characteristics, and screening risks to address this evidence gap and inform recommendations by the USPSTF and others (12-14).

To date, most models of CRC screening have not incorporated the serrated pathway, and most model estimates do not reflect the substantial uncertainty in assumptions about the natural history from normal colon to invasive CRC (15). We developed a new decision-analytic model that overcomes these limitations. We validated the model against published data and used it to assess the comparative effectiveness, cost-effectiveness, and burden–benefit balance of current and emerging stool-based CRC screening tests and colonoscopy.

## Methods

### Model

How well decision-analytic models reflect reality depends on a model’s *structure*, which constrains how the modeled disease may develop over time, across individuals, and subject to interventions (eg, a discrete number of health states vs a continuous representation); and *parameter* values, which determine how the disease behaves (eg, how fast adenomas progress). For a given structure, multiple sets of parameters may be consistent with the 95% confidence interval around real-world observations, and contrasting parameter sets could potentially yield different answers to critical questions such as how to screen most cost-effectively.

**Figure 1.**
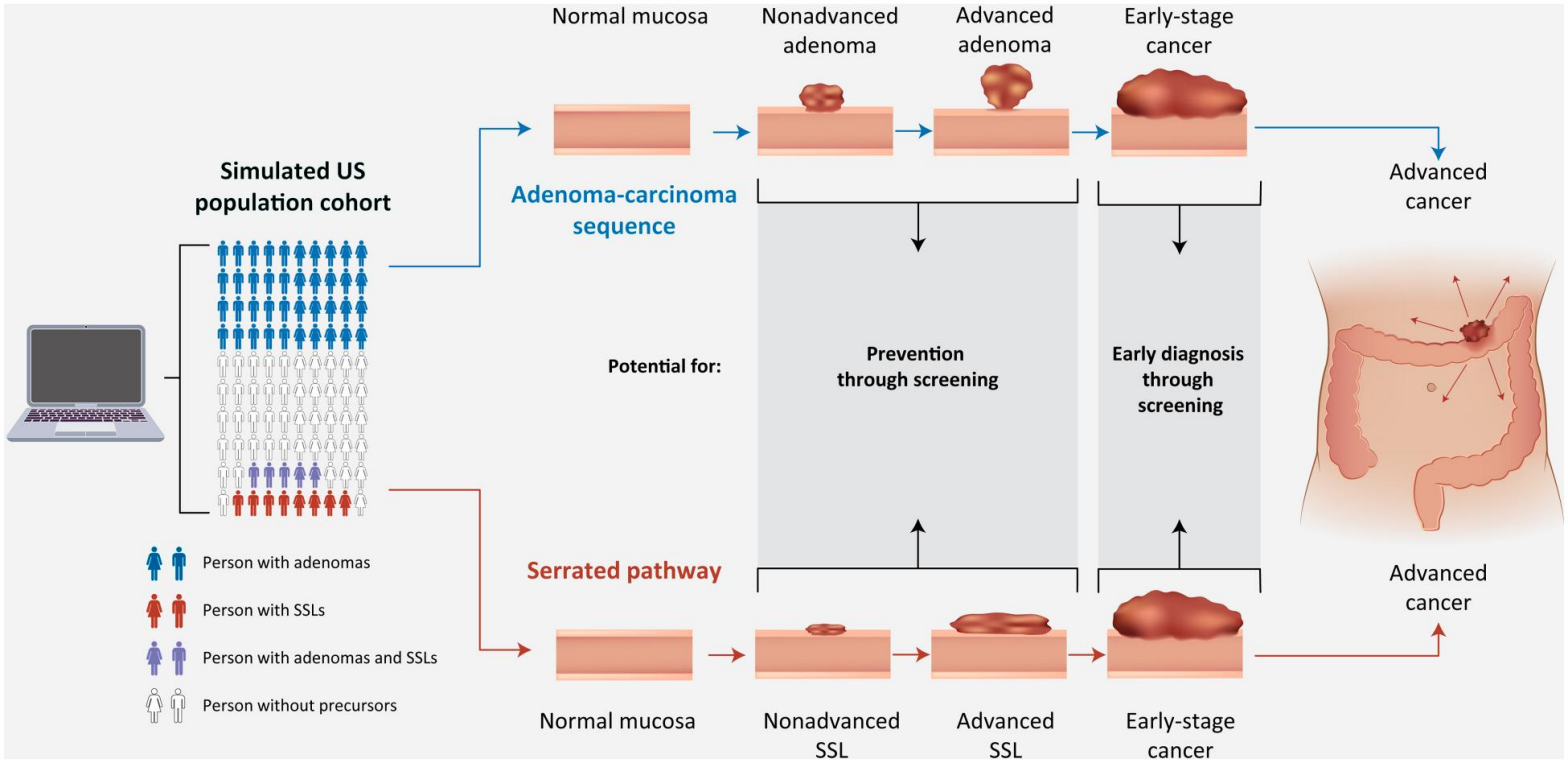
Adenoma and serrated pathway (ANSER) microsimulation model of colorectal cancer. ANSER simulates a cohort of US adults, some of whom develop adenomas, sessile serrated lesions (SSLs), or both during their lifetime. Precancerous lesions cycle through nonadvanced, advanced, early cancer, and advanced cancer stages at different rates. Screening may prevent colorectal cancer deaths by detection and removal of precancerous lesions, or by earlier diagnosis.

We developed the Adenoma aNd SERrated pathway (ANSER) microsimulation model to innovate in two key respects: 1) ANSER simulates the natural history of CRC through the adenoma or serrated pathway for a US birth cohort, reflecting up-to-date knowledge about CRC precursor epidemiology (Figure 1, Supplementary Figure 1, available online), and 2) every output from ANSER includes 95% uncertainty intervals (UIs) that incorporate the uncertainty in source data, instead of being only a point estimate. ANSER’s principal calibration was anchored in published observations of precursor prevalence, features, and CRC incidence by age (Supplementary Figures 2-4, available online, evidence successful calibration) (16,17). We derived a family of transition parameter sets between health states consistent with 95% confidence intervals around observed precursor and CRC data. Each simulation considers the family of 100 sets selected to reflect the uncertainty in source data. In addition, each simulation considers variability in other model inputs, directly informed by data (eg, test performance and cost). Thus, ANSER yields results with outcome distributions that account for the uncertainty in the data sources, instead of using single point estimates.

ANSER’s structure, data sources, calibration, uncertainty, and validation are detailed in the Supplementary Materials (Supplementary Methods, Supplementary Figure 1-6, Supplementary Tables 1-3, available online).

### Screening test attributes

Current USPSTF guidelines recommend colonoscopy every 10 years, sDNA-FIT every 1 to 3 years, and FIT every year, for ages 45-75 years, and other less common options (10). Colonoscopy allows direct visualization and removal of detected lesions; stool tests require colonoscopy follow-up. Colonoscopy is the most sensitive but also the most invasive and costly (18). FIT with a positivity cutoff of 20 µg Hb/g stool (FIT-20) has acceptable CRC sensitivity (19), but lowering its cutoff to 10 µg Hb/g stool (FIT-10) improves CRC and adenoma sensitivity (20). sDNA-FIT has better one-time sensitivities for CRC, advanced adenomas, and SSLs, and lower specificity than FIT-20, but is similar to FIT-10 except for its higher SSL sensitivity (19). In preliminary data, an emerging version (sDNA-FIT2.0) has improved sensitivity and specificity (21). Full test characteristics are summarized in Table 1.

**Table 1.** Assumed test performance including 95% confidence intervals for uncertainty analysis

Most advanced lesion	**Colonoscopy** ^b^	**sDNA-FIT** ^c^	**FIT-20** ^d^	**sDNA-FIT2.0** ^e^	**FIT-10** ^f^
No lesion (specificity)	100 (-)	89.8 (88.9 to 90.7)	96.4 (95.8 to 96.9)	92.3 (89.2 to 94.8)	90 (87 to 93)
Nonadvanced adenoma^a^	69 (53 to 82)/81 (72 to 88)	17.2 (15.9 to 18.6)	7.6 (6.7 to 8.6)	15.9 (10.8 to 22.2)	15 (10 to 20)
Advanced adenoma	90 (80 to 97)	42.4 (38.9 to 46.0)	23.8 (20.8 to 27.0)	57.1 (46.8 to 67.1)	40 (33 to 47)
Nonadvanced SSL^a^	69 (53 to 82)/81 (72 to 88)	17.2 (15.9 to 18.6)	3.6 (3.1 to 4.2)	15.9 (10.8 to 22.2)	10 (7 to 13)
Advanced SSL	90 (80 to 97)	42.4 (38.9 to 46.0)	3.6 (3.1 to 4.2)	57.1 (46.8 to 67.1)	10 (7 to 13)
Colorectal cancer	91 (84 to 96)	92.3 (83.0 to 97.5)	73.8 (61.5 to 84.0)	95.5 (89.9 to 98.5)	91 (84 to 95)

a In the model, nonadvanced lesions include 1 to 5 mm and 6 to 9 mm lesions, with differing associated sensitivities for colonoscopy. FIT-X = fecal immunochemical test with cutoff X µg hemoglobin per g stool; sDNA-FIT = stool-DNA/FIT; SSL = sessile serrated lesion.

b For colonoscopy, sensitivity is applied at the lesion level, not the patient level, given the most advanced lesion present. Per-lesion sensitivities are from Zhao et al. (18), Design A (conventional colonoscopy followed by enhanced colonoscopy). No size or stage-specific estimates were available for SSLs, but given similar pooled sensitivity vs adenomas, we assumed similar sensitivity across sizes/stages for both etiologies; lower sensitivity for SSLs was evaluated in sensitivity analysis. For cancer, we used observed sensitivity for large adenomas.

c From Imperiale et al. (19), who reported combined estimates for adenomas and serrated lesions, except for advanced lesions, for which sensitivity was similar by etiology.

d From Imperiale et al. (19), who reported combined estimates for adenomas and serrated lesions, except for advanced lesions, for which plotted estimates suggested no sensitivity for serrated lesions.

e Evaluated in sensitivity analysis. Estimates from Kisiel et al. (21), who verified discoveries from a case-control study in a set of prospectively collected stool samples mimicking a screening population.

f Evaluated in sensitivity analysis. Estimates from a meta-analysis by Imperiale et al. (20). Sensitivity for SSLs was assumed to equal 1—the specificity.

### Analysis

#### Clinical and economic outcomes

Key outcomes included lifetime CRC incidence, CRC mortality, and life-years gained per 1000 45-year-olds, and the quality-adjusted life-years (QALYs) gained, colonoscopies required, and health-sector costs per person, all including 95% UIs. QALYs and costs were discounted by 3% per year.

#### Validation of the impact of screening

ANSERs predicted effects of screening were validated against randomized controlled trial results of the Minnesota Colorectal Cancer Control Study of annual stool-based screening (5) and the UK Flexible Sigmoidoscopy Study (22). After simulating the trials’ designs and participation patterns (see Supplementary Methods, available online), we compared the model-predicted and trial-observed CRC incidence and mortality curves over time for each study arm. We considered ANSER to be validated if the mean predicted relative risk reductions for screening fell within the 95% CIs of the trial-observed data.

#### Primary analysis

In the primary analysis, we evaluated recommended colonoscopy and stool-based strategies, ie, colonoscopy every 10 years, FIT-20 every year, and sDNA-FIT every 3 years, with colonoscopy surveillance for patients with detected adenomas or SSLs per US guidelines (23). Adherence to screening, follow-up of positive stool tests, and surveillance were assumed to be 100%. Strategies were compared using current-standard decision-analytic and cost-effectiveness modeling approaches (24). Each strategy was evaluated 11 000 times using different model parameter sets (permutations) reflecting observed 95% confidence intervals (CIs) or input uncertainty (Supplementary Table 2, available online). Modeled 95% UIs were derived as the 2.5-97.5 percentile range across iterations. Optimal strategies were determined based on 3 criteria: effectiveness, cost-effectiveness, and burden–benefit tradeoffs. For effectiveness, we focused on CRC deaths averted. Because there is no single agreed-upon cost- or burden-acceptance threshold in the United States, the last 2 outcomes were evaluated using cost-effectiveness acceptability curves and frontiers (15): the curves represent the probability a strategy is optimal at different cost- or burden-acceptance thresholds (or “willingness-to-pay” thresholds, eg, $100 000/QALY gained); the frontier highlights the strategy with the highest expected benefit at each threshold. For burden–benefit analysis, we evaluated required colonoscopies/life-year gained, similar to the USPSTF (12).

#### Sensitivity analyses

In sensitivity analyses, we evaluated sDNA-FIT2.0 and FIT-10, in addition to the currently available tests in the United States, and considered stool DNA at 1- and 3-year intervals. We compared sDNA-FIT and FIT-10 at annual intervals to isolate the impact of the higher SSL sensitivity of sDNA-FIT.

The principal sensitivity analyses explored the plausible range of CRCs arising from SSLs. Whereas the base case assumed stable CRC incidence over time and the same progression rates to CRC from adenomas and SSLs (16), in sensitivity analysis we recalibrated the model to evaluate: 1) twofold increased SSL prevalence and colonoscopy miss rates for SSLs vs adenomas, on the basis of our systematic review suggesting higher SSL detection rates in high-quality colonoscopy or with more liberal histopathologic criteria (16); 2) twofold increased progression of SSLs vs adenomas to CRC, considering the higher proportion of interval CRCs in the right-sided colon where SSLs are more common (25); 3) a combination of twofold increased prevalence and threefold increased progression reflecting a plausible upper bound for the fraction of CRC arising from SSLs; and 4) 1.54-fold increased precursor and CRC incidence vs the base case, extrapolating rising incidence in early-onset CRC (26,27).

### Software and hardware

ANSER was programmed in R version 4.0.2 (28), and used Dampack for cost-effectiveness and burden-to-benefit analysis (29). Analyses were conducted on Sherlock 2.0, Stanford University’s high-performance computing cluster of 44 032 cores. Estimated computing cost was in the magnitude of 10^4^ core-hours.

### Institutional review board

No institutional review was required for this modeling work.

## Results

### Model validation

**Figure 2.**
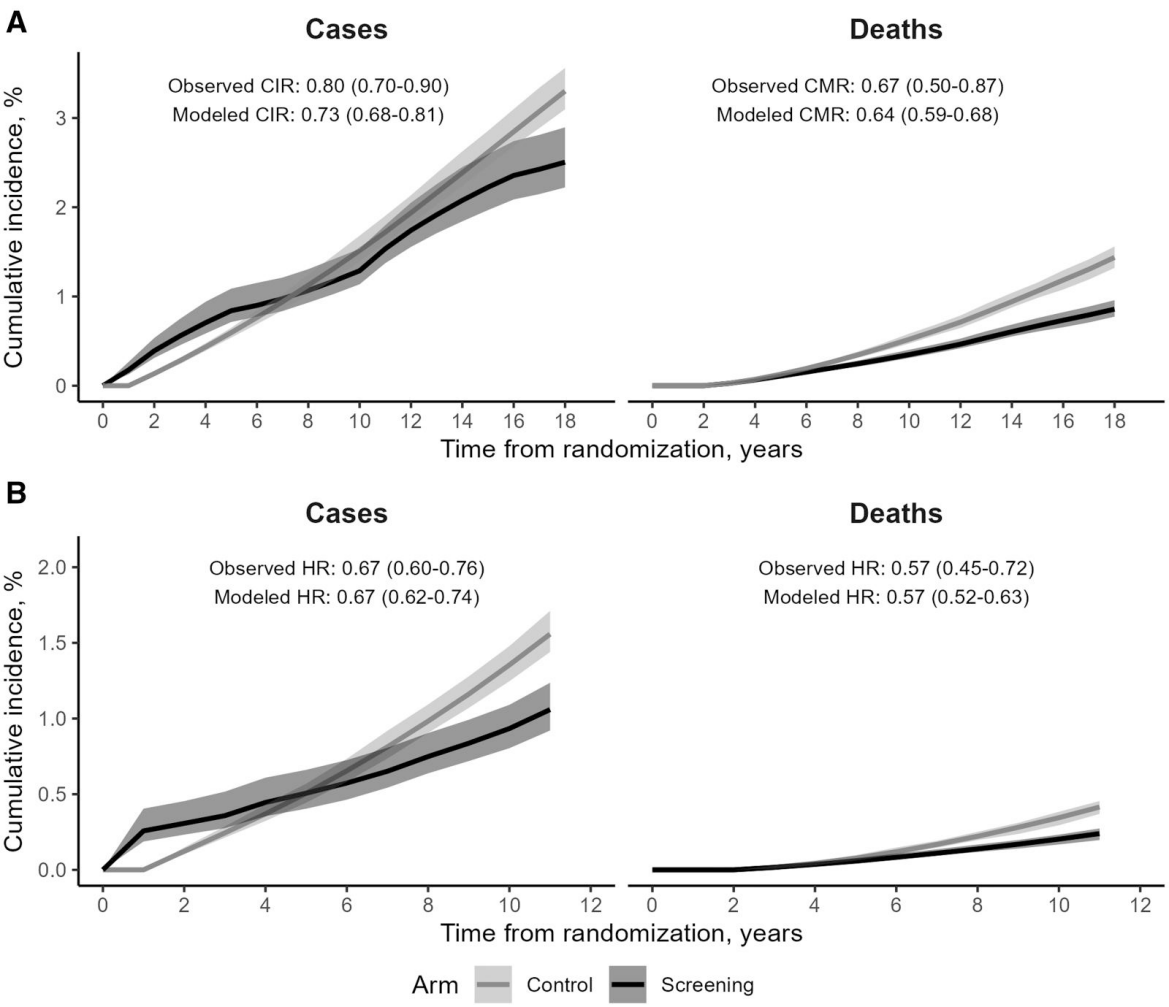
Validation of ANSER’s predictions against the Minnesota Colorectal Cancer Control Study (**A**) and the UK Flexible Sigmoidoscopy Trial (**B**). Figures show outcomes of annual guaiac-based fecal occult blood testing vs no screening (**panel A**) and once-only sigmoidoscopy vs no screening (**panel B**). Lines reflect simulated means, ribbons 95% uncertainty intervals. CIR = cumulative incidence ratio, CMR = cumulative mortality ratio, HR = hazard ratio. The model is considered valid because modeled relative risks fall within 95% confidence intervals around the observed estimates.

ANSER’s predictions matched the CRC incidence and mortality curves, and the reductions from screening, observed in the Minnesota Colorectal Cancer Control Study and the UK Flexible Sigmoidoscopy Study (relative CRC mortality for screening and control arms in ANSER vs trials: 0.64 [95% UI = 0.59-0.68] vs 0.67 [95% CI = 0.50 to 0.87], and 0.57 [95% UI = 0.52-0.63] vs 0.57 [95% CI = 0.45 to 0.72], respectively) (Figure 2).

### Primary analysis: Effectiveness

Without screening, among 1000 simulated 45-year-olds, 62.5 (95% UI = 58.8-66.3) developed CRC and 24.1 (95% UI = 22.5-25.7) died from CRC during an average 35.9 life-years (95% UI = 35.8-36.0).

**Figure 3.**
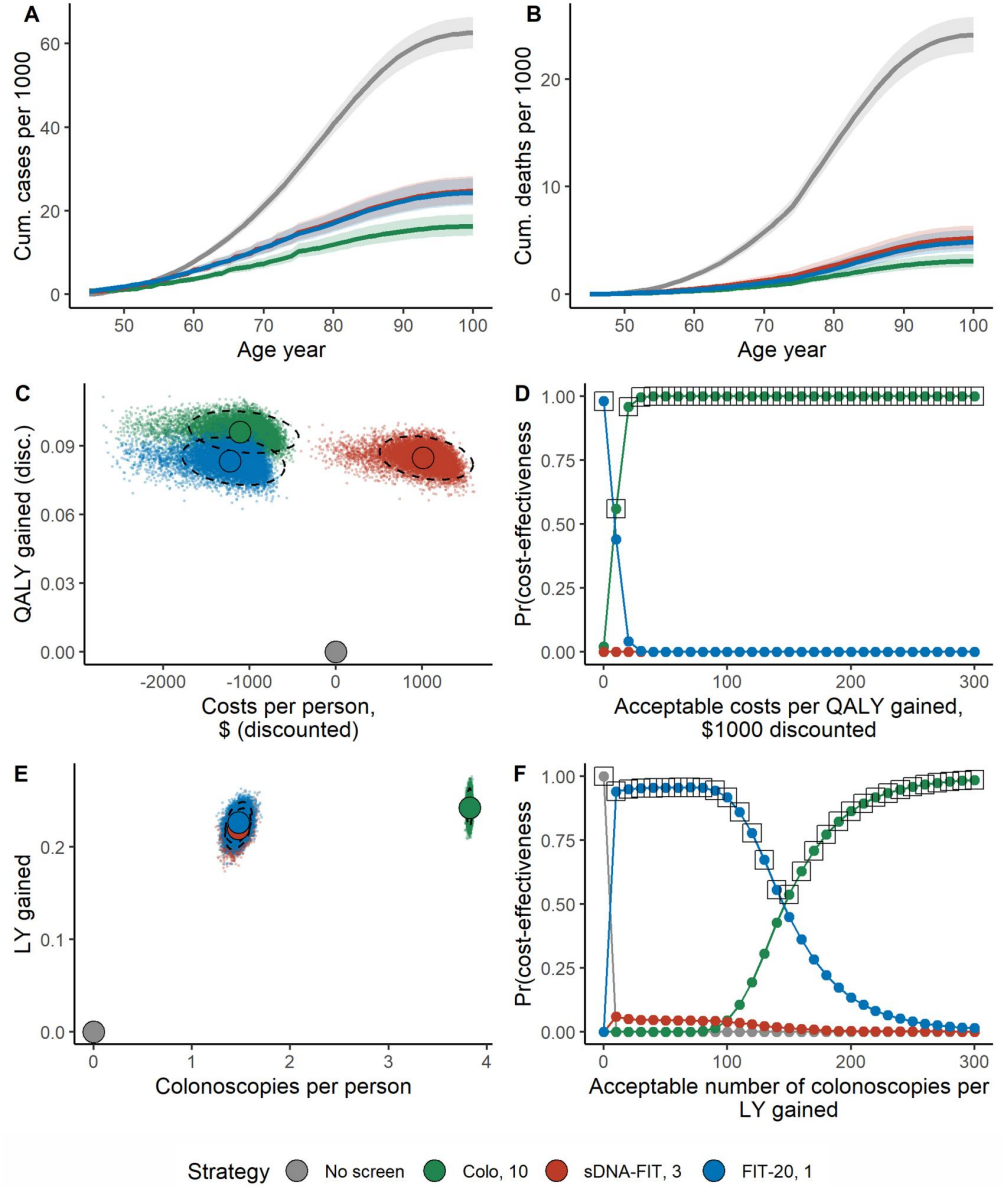
Lifetime effectiveness, cost-effectiveness, and burden–benefit of screening. Figures compare selected screening strategies in terms of simulated lifetime colorectal cancer incidence (**panel A**) and mortality (**panel B**), QALYs gained over net screening costs and cost-effectiveness acceptability (**panel C, D**), and life-years gained over required colonoscopies and burden-effectiveness acceptability (**panel E, F**). Lines represent means across model iterations, ribbons 95% uncertainty intervals (**panel A, B**); dots single iterations, solid circles means, dashed ellipses 95% uncertainty ranges (**panel C, E**); lines strategies’ cost-effectiveness probabilities at a given cost or burden-acceptance threshold, squares strategies with the highest expected benefit (**panel D, F**). Strategies are denoted as “Test, Interval,” where Colo = colonoscopy, FIT-X = fecal immunochemical test with a cutoff equal to X, sDNA-FIT = stool-DNA/FIT, LY = life-year, QALY = quality-adjusted life-year.

The screening strategies reduced CRC incidence by a mean 61%-74%, and CRC mortality by a mean 78%-87%, depending on the strategy (Figure 3A-B, Table 2). Colonoscopy produced the greatest mean clinical benefit, averting 46.3 (95% UI = 46.2-50.0) CRCs and 21.0 (95% UI = 19.5-22.5) CRC deaths per 1000 persons. In contrast, sDNA-FIT every 3 years averted 37.9 (95% UI = 34.1-41.6) CRCs and 18.9 (95% UI = 17.3-20.5) CRC deaths and FIT-20 averted 38.3 (95% UI = 34.4-42.0) CRCs and 19.3 (95% UI = 17.6-20.8) CRC deaths per 1000 persons.

**Table 2.** Lifetime effectiveness, cost, and burden of screening

Outcome	Strategy		
(95% UI)	Colonoscopy every 10 years	sDNA-FIT every 3 years	FIT-20 every 1 year
CRC cases averted per 1000 persons	46.3 (42.6-50)	37.9 (34.1-41.6)	38.3 (34.4-42)
% reduction vs no screening	74.0 (69.6-77.3)	60.6 (55.5-64.9)	61.2 (56.1-65.6)
CRC deaths averted per 1000 persons	21.0 (19.5-22.5)	18.9 (17.3-20.5)	19.3 (17.6-20.8)
% reduction vs no screening	87.3 (84.5-89.2)	78.5 (73.8-82)	80 (75.4-83.1)
Life-years gained per 1000 persons	242 (223-262)	219 (198-239)	226 (205-246)
QALYs gained per person^a^	0.096 (0.088-0.105)	0.085 (0.076-0.093)	0.083 (0.074-0.093)
Incremental cost per person, $^a^	−1100 (−2000 to −700)	1000 (300-1400)	−1200 (−2000 to −800)
Cost per QALY gained vs no screening, $^a^	Colonoscopy dominates	12 100 (3300-17 700)	FIT-20 dominates
Cost per QALY gained vs FIT-20, $^a^^,^^b^	9300 (500-21 900)	792 400 (FIT-20 dominates to cost of 12.8 million)	–
Number of stool tests per person	–	7.0 (6.8-7.3)	18.7 (17.9-19.6)
Number of colonoscopies per person	3.83 (3.8-3.85)	1.47 (1.35-1.59)	1.47 (1.35-1.6)

a Marked outcomes are discounted by 3% per year. CRC = colorectal cancer; FIT-X = fecal immunochemical test with cutoff X; sDNA-FIT = stool-DNA/FIT; QALY = quality-adjusted life-year; UI = uncertainty interval.

b Median reported for costs per QALY gained vs FIT-20 instead of mean, which was strongly influenced by outliers.

The impact on CRC outcomes translated to a mean 219-242 life-years gained per 1000 persons, or 0.083-0.096 discounted QALYs gained per person screened, depending on the strategy (Table 2). The distribution clouds for gains in QALYs largely overlapped for stool tests (Figure 3C) but were more disparate for colonoscopy. Despite some overlap, colonoscopy averted the most CRC deaths in 100% of evaluations, implying correlated outcomes between strategies and robust effect differences (Table 3).

**Table 3.** Optimal strategies across sensitivity analyses^a^

Outcome	Scenario				
(95% UI)	Base case	Increased SSL prevalence	Increased SSL progression	Increased SSL prevalence & progression	Increased overall CRC incidence
Fraction of CRC arising from SSLs, %	11.8 (8.1-15.8)	16.6 (13.0-20.3)	20.1 (14.6-25.5)	23.1 (18.8-26.8)	11.7 (8.6-14.7)
Maximum CRC mortality reduction, % of model iterations					
Colonoscopy, every 10 years	100	100	100	100	100
sDNA-FIT, every 3 years	0	0	0	0	0
FIT-20, every year	0	0	0	0	0
Maximum QALY s.t. $100k/QALY gained, % of model iterations					
Colonoscopy, every 10 years	100	100	100	100	86.7
sDNA-FIT, every 3 years	0	0	0	0	1.5
FIT-20, every year	0	0	0	0	11.8
Maximum life-years s.t. 100 colonoscopies/LYG, % of model iterations					
Colonoscopy, every 10 years	4.4	1.8	11.0	2.1	45.3
sDNA-FIT, every 3 years	3.9	1.3	11.6	2.0	6.7
FIT-20, every year	91.7	96.9	77.4	95.9	48.0
Cost per QALY gained, sDNA-FIT vs FIT-20, $	792 400 (FIT-20 dominates to 12.8 million)	722 700 (FIT-20 dominates to 17.1 million)	701 600 (FIT-20 dominates to 8.9 million)	750 000 (FIT-20 dominates to 15.6 million)	FIT-20 dominates (FIT-20 dominates to 10.7 million)

a CRC = colorectal cancer; FIT-X = fecal immunochemical test with cutoff X; sDNA-FIT = stool-DNA/FIT; SSLs = sessile serrated lesions; QALY = quality-adjusted life-year; s.t. = subject to; UI = uncertainty interval.

### Primary analysis: Cost-effectiveness

Higher total costs/person were evident in the outcome distribution clouds for sDNA-FIT vs colonoscopy or FIT (Table 2, Figure 3C). Colonoscopy and FIT-20 were net cost-saving and therefore dominant vs no screening (Table 2). In contrast, sDNA-FIT cost $1000 per person screened, for a cost/QALY gained vs no screening of $12 100 (95% UI = $3300-17 700). sDNA-FIT strategies cost more than $500 000 per QALY gained vs FIT-20.

The optimal strategy on the basis of cost-effectiveness was FIT-20 at cost-acceptance thresholds less than $10 000/QALY gained, and colonoscopy at higher thresholds (Figure 3D). At a cost-effectiveness threshold of $100 000 per QALY gained, the preferred strategy was colonoscopy in 100% of iterations (Table 3).

### Primary analysis: Burden-to-benefit tradeoffs

The outcome distribution clouds also clearly separated on colonoscopy burden (Table 2, Figure 3E). Screening required 3.9 (95% UI = 3.8-3.9) colonoscopies/person in colonoscopy screening, and a number of 1.5 (95% UI = 1.4-1.6) in sDNA-FIT and FIT-20 (Table 2). Stool-based screening required 7.0 (95% UI = 6.8-7.3) stool tests/person for sDNA-FIT every 3 years, and 18.7 tests/person (95% UI = 17.9-19.6) for FIT-20 (Table 2).

FIT-20 was the optimal strategy in terms of colonoscopies/life-years gained (Figure 3F) up to a threshold of 150 colonoscopies per life-year gained, above which colonoscopy was optimal.

### Sensitivity analysis: Inclusion of alternative stool tests

In sensitivity analysis, FIT-10 every year, sDNA-FIT every year, and sDNA-FIT2.0 every 1-3 years produced intermediate benefits compared with FIT-20 and colonoscopy screening (Supplementary Table 4, Supplementary Figure 7, available online). Colonoscopy remained optimal in effectiveness and cost-effectiveness for thresholds at or above $10 000/QALY gained. Annual sDNA-FIT and sDNA-FIT2.0 were optimal in burden–benefit but cost more than $3000 per person (Supplementary Table 5, available online). However, sDNA-FIT2.0 every 3 years was cost-effective in some model iterations.

Annual sDNA-FIT reduced CRC incidence by 70.0% (95% UI = 65.2%-73.6%) and CRC mortality by 85.2% (95% UI = 81.7%-87.5%). By comparison, annual FIT-10 reduced CRC incidence by 68.0% (95% CI = 62.8% to 72.3%) and CRC mortality by 84.2% (95% UI = 80.3%-86.8%).

### Sensitivity analysis: Higher fraction of CRCs from SSLs and higher overall CRC incidence

The fraction of CRCs arising from SSLs varied across scenarios (Table 3), from 11.8% (95% UI = 8.1%-15.8%) in the base case to 16.6% (95% UI = 13.0%-20.3%) with increased SSL prevalence, 20.1% (95% UI = 14.6%-25.5%) with increased progression of SSLs vs adenomas, and 23.1% (95% UI = 18.8%-26.8%) with increased SSL prevalence and risk; the fraction was almost unchanged with increased background CRC incidence. As the fraction of CRCs arising from SSLs increased, the rank-ordering of strategies’ comparative effectiveness did not change substantially; colonoscopy remained most cost-effective, and FIT-20 was optimal in burden–benefit (Table 3, Supplementary Figures 8-11, available online). sDNA-FIT did not become cost-effective vs FIT-20. With a higher background CRC incidence, the burden–benefit ratio of colonoscopy improved, but FIT-20 remained optimal in that respect.

## Discussion

We developed a novel CRC screening decision-analytic model (ANSER) with two distinguishing features: inclusion of the serrated pathway in addition to the adenoma pathway, and consideration of the substantial uncertainty related to natural history parameters. We used this model to address questions regarding the comparative effectiveness, cost-effectiveness, and burden–benefit balance of screening strategies that differ in their ability to detect adenomas, SSLs, and CRCs.

Our primary results highlight colonoscopy every 10 years as the most effective and cost-effective CRC screening strategy when both the adenoma and serrated pathways are considered. By comparison, the stool-based strategies produced benefits amounting to more than 80% of the CRC incidence reduction and more than 90% of the mortality benefit compared with colonoscopy.

The tests’ outcome distributions showed clear separations for costs. Although all strategies were cost-effective vs no screening, colonoscopy and FIT emerged as the cost-efficient strategies. Our modeling suggests that colonoscopy’s ability to detect SSLs is significant enough to make colonoscopy cost-effective vs FIT at low cost-acceptance thresholds, a finding that is in contrast to previous models in which SSLs are not considered, and in which colonoscopy is often found to be more costly vs FIT (30,31). In contrast, our modeling suggests that sDNA-FIT’s SSL sensitivity, which is often touted as a key advantage over FIT, and which may equalize its effectiveness to annual FIT, is not enough to make sDNA-FIT cost-effective vs FIT.

Stool-based strategies required 1-2 colonoscopies/person over a lifetime, versus almost 4/person in colonoscopy screening, but they required an average of 7-19 stool tests/person. Similar to USPSTF-commissioned modeling (12), we found annual FIT screening to provide a better balance of colonoscopies per life-year gained than the current sDNA-FIT.

We are aware of 3 CRC screening decision-analytic models that included the serrated pathway. These models were developed for the Netherlands (32), Germany (33), and Australia (34). They did not consider the screening strategies most relevant to the United States or holistic uncertainties in other factors such as test performance, and they evaluated most scenarios at an arbitrary 15% of CRCs arising from serrated lesions. We varied all relevant assumptions based on the ranges in source data and explored the optimal strategies based on clinical effectiveness, cost-effectiveness, or burden–benefit balance to align with US policy and practice (24).

We acknowledge the limitation that we evaluated strategies with 100% screening participation. Participation may be the most important driver of screening effectiveness (35,36). A given participation rate is probably not a constitutive attribute of a screening test; rather, patterns of participation differ substantially between settings and patients depending on deployment strategies for a specific test, access and availability of clinical resources, and patient preferences. We elected to estimate the expected benefit from screening when patients follow screening recommendations, which is standard practice to inform guidelines (12,14). Benefits and costs of screening at lower levels of participation to any strategy (but with consistent per-cycle participation over time) may be approximated by proportionally scaling the results presented here. In the future, new modalities such as blood tests could improve overall patient participation (37), but these modalities are not yet widely available. Future modeling efforts can focus on novel tests or hybrid strategies that shift between tests over the life-course.

Another limitation is that hyperplastic polyps were not simulated. According to observational data, these lesions may be detected in isolation from adenomas or SSLs in up to 10% of average-risk adults (38). Because the risk of complications after removal of small hyperplastic polyps is negligible and because subsequent screening intervals are the same as after normal colonoscopy, hyperplastic polyps mainly affect pathological workup costs. On average, these additional colonoscopy costs are within 1 standard deviation of the variation already considered in our analyses and therefore unlikely to change conclusions.

Although we successfully calibrated ANSER’s natural history to observed data (Supplementary Figures 2-4, available online) and propagated data uncertainty through 95% UIs, some uncertainties around the model’s assumptions remain, similar to any alternative model. Relevant factors such as the perceived prevalence and progression of SSLs, background CRC incidence and the relationship to comorbidity, test performance, and health-care costs are all subject to change, and our assumptions were necessarily based on observations from the past. ANSER’s predictions were compared against results of other models and randomized clinical trials of stool-based and sigmoidoscopy screening. Without adjusting the model’s structure or parameters to match the trials’ observations, ANSER reproduced key study outcomes. Relevant assumptions were varied in base-case and sensitivity analyses, with relatively stable outcomes, increasing confidence in the model’s suggested conclusions.

There is particular uncertainty around the prevalence and progression of SSLs. We modeled alternative combinations of colonoscopy miss rates of SSLs and estimates of prevalence in the natural history model, which, combined, are consistent with the observed SSL detection rates reported in the literature. The assumed miss rate of SSLs for our base-case analysis was similar to that for adenomas, at approximately 1 in 4, based on a meta-analysis of tandem colonoscopy studies (18). This assumed equivalence may seem inconsistent with some physicians’ intuition and with the range in SSL detection for average vs high-performing endoscopists (16), but it is consistent with data showing similar variation and high correlation in physician’s adenoma vs serrated lesion detection rates (39). Recognizing the uncertainty and significance of these assumptions, we included sensitivity analyses that capture scenarios with higher assumed miss rates and prevalence of SSLs, none of which affected our overall conclusions.

Our results have implications for CRC screening policy. First, all evaluated screening strategies are likely to offer a high degree of protection against CRC, while differing meaningfully in tradeoffs of cost-effectiveness and burden–benefit. Health systems and patients may have different preferences regarding these tradeoffs. Our findings support US recommendations to offer patients an informed choice between screening options. Second, the relatively higher sensitivity of sDNA-FIT for advanced adenomas and SSLs makes sDNA-FIT every 3 years approximately equivalent to annual FIT in terms of CRC clinical benefit. The tradeoffs between these strategies are the test process and interval, colonoscopy referral rate, and test cost. Third, although cost-effectiveness estimates are not considered by the USPSTF, health systems seek to limit per-patient costs while improving patients’ outcomes and experiences (Institute of Medicine’s “Triple Aim”). Some recommended strategies may not be feasible for some payers unless dramatic differences in population-level participation rates can be demonstrated for the more versus the less costly strategies. The USPSTF considers sDNA-FIT annually, but in practice it is used every 3 years. Finally, although consideration of the serrated pathway did not result in sDNA-FIT as the preferred stool-based test in our base-case scenarios, the emerging sDNA-FIT2.0 was the preferred stool-based test in a substantial fraction of model iterations in sensitivity analysis due to a higher specificity.

In conclusion, ANSER addresses questions regarding the comparative effectiveness, cost-effectiveness, and burden–benefit balance of alternative CRC screening strategies, accounting for the serrated and adenoma pathway, and for the substantial uncertainty in CRC natural history. Our estimates of clinical effectiveness support US guidelines to offer patients a menu of screening options, despite varying levels of SSL detection. Our economic estimates highlight the profound influence of test cost when participation rates are comparable across strategies and can inform decision-making bodies considering cost-effectiveness in their deliberations.

## Supplementary Material

pkae077_Supplementary_Data

## Data Availability

Requests for model source data can be directed to rmeester@stanford.edu.
