# Supplementary material for: Impact of the serrated pathway on the simulated comparative effectiveness of colorectal cancer screening tests
Source: JNCI Cancer Spectr. 2024 Sep 6;8(5):pkae077. doi: 10.1093/jncics/pkae077 (PMC11470154; doi:10.1093/jncics/pkae077)
Supplement: pkae077_Supplementary_Data [file pkae077_supplementary_data.pdf]

Impact of the serrated pathway on the simulated comparative effectiveness of colorectal cancer screening tests

Supplementary Materials

Reinier G.S. Meester PhD, Uri Ladabaum MD MS

Stanford University School of Medicine

Division of Gastroenterology and Hepatology

Contents

Supplementary Methods 3
Model overview 3
Model architecture 3
Model assumptions and data sources 5
Model calibration 9
Model uncertainty 12
Cross-model validation 13
External model validation 15
References 17
Supplementary Figure 1. Natural history of colorectal cancer as simulated by ANSER. 21
Supplementary Figure 2. Calibrated precursor prevalence by age and sex. 22
Supplementary Figure 3. Calibrated precursor features. 23
Supplementary Figure 4. Calibrated CRC incidence by age, sex and stage. 24
Supplementary Figure 5a. Observed CRC survival by age, stage and location in women. 25
Supplementary Figure 5b. Observed CRC survival by age, stage and location in men. 26
Supplementary Figure 6. Variation in CRC-related treatment, procedures, and tests. 27
Supplementary Figure 7. Lifetime effectiveness, cost-effectiveness and burden-benefit of all strategies. 28
Supplementary Figure 8. Lifetime effectiveness, cost-effectiveness and burden-benefit of screening strategies: *high SSL prevalence* scenario. 29
Supplementary Figure 9. Lifetime effectiveness, cost-effectiveness and burden-benefit of screening strategies: *high SSL risk* scenario. 30
Supplementary Figure 10. Lifetime effectiveness, cost-effectiveness and burden-benefit of screening strategies: *high SSL prevalence and risk* scenario. 31
Supplementary Figure 11. Lifetime effectiveness, cost-effectiveness and burden-benefit of screening strategies: *increased background incidence reflecting early-onset trends*. 32
Supplementary Table 1. ANSER's natural history model form, parameters, and data sources. 33
Supplementary Table 2. ANSER parameters including 95%CIs for uncertainty analysis. 34
Supplementary Table 3. Cross-model comparison in outcomes. 38
Supplementary Table 4. Lifetime effectiveness, burden and cost for all evaluated strategies. 40
Supplementary Table 5. Optimal among all evaluated strategies in sensitivity analyses. 42

Supplementary Methods

Model overview

The adenoma and serrated pathway (ANSER) model simulates a U.S. population cohort, one individual at a time, with stochastic variation in the development, growth and progression of precancerous lesions and colorectal cancer (CRC) through two distinct etiologic pathways. Model parameters are informed by real-world observations, where needed using calibration. The model's predicted effects of interventions are compared against other models and validated against randomized controlled trial data. ANSERs predictions account for input uncertainty by considering, for each analysis, a family of parameter sets consistent with observed source data.

Each of these features are described in more detail below.

Model architecture

ANSER is an microsimulation model programmed in R statistical software.[1] It combines features of Markov models and discrete-event simulation, to reduce the computational burden from random number generation (for details see **Supplementary Table 1**).[2] Agents are characterized by a death age sampled using U.S. life tables, and by the different rates of development of CRC through the two etiologic pathways causing potential earlier death: the adenoma-carcinoma sequence and the serrated pathway.

The *adenoma-carcinoma sequence* has long been recognized as the main etiologic pathway to CRC.[3] Features associated with progression to CRC include large size, villous histology, and presence of high-grade dysplasia. We use size as a proxy for all the above, as villous histology and high-grade dysplasia strongly correlate with size[4,5] and are not as well reported in autopsy studies from the pre-screening era, which served as our main adenoma data source (see Model calibration).

The *serrated pathway* to CRC was recognized more recently, and there is variation in literature on how it is characterized.[6] The WHO 5th manual of classification of

tumors distinguishes four types of serrated lesions:[7] sessile serrated lesions (SSLs), traditional serrated adenomas (TSAs), hyperplastic polyps (HPs), and mixed polyps combining adenoma and serrated histologies. It is generally accepted that among pure serrated lesions, SSLs and TSAs comprise the main clinically relevant serrated lesions, whereas HPs are generally innocuous. However, SSLs can be hard to distinguish pathologically from HPs, which is why some experts have suggested to include large and/or proximal HPs among clinically significant serrated lesions.[8] For simplicity, we used SSL terminology and data, since our review indicated that TSAs are extremely rare (0.3% prevalence).[6] However, we varied assumptions in sensitivity analysis to account for more liberal definitions. For SSLs, size and presence of any cytologic dysplasia are known markers for progression to CRC. As for adenomas, we model size alone considering the more limited availability of data on SSLs with dysplasia.[6]

ANSER's distinguished health states therefore include (**Supplementary Figure 1**): healthy (normal colorectal mucosa), nonadvanced (0-5 and 6-9 mm diameter) and advanced (≥10 mm diameter) adenomas or SSLs, preclinical CRC progressing through stage I, II, III and IV, clinical CRC diagnosed in either of those stages, and CRC or other-cause death (competing risks), whichever comes first. The time of other-cause death is first sampled for each simulated individual based on U.S. life tables. Then, the times of precursor lesion incidence or formation using a data-informed hazard rate (see **Model calibration**), allowing multiple synchronous lesions to exist at any time. A Markov process updates patient health states at annual cycles with transition parameters calibrated to other source data, including observed CRC incidence by age, sex and stage. Outcomes are corrected for discontinuity using Simpson's 1/3rd rule.[9]

Although the model was designed with the flexibility to include complex correlation structures and heterogeneity, by default, no correlation was assumed between CRC development and competing risks, between adenoma and SSL formation, or growth and progression. Also, no individual-level heterogeneity was assumed in the (mean) rates of polyp formation. The latter means that although there is stochasticity and variation in patient outcomes, these are the result of drawing from the same underlying probability distributions by sex.

In the model, screening is superimposed on the natural history of CRC and may prevent CRC death through precursor detection and removal or earlier CRC diagnosis. A screening test's efficacy depends on the sensitivity for CRC and precursors, and in case of noninvasive tests, the timing and quality of colonoscopy follow-up. After polypectomy, some rate of lesion recurrence is assumed based on surveillance data.

Model assumptions and data sources

Multiple data sources were used to inform ANSER's assumptions. Assumptions were varied in uncertainty analysis. An overview of all model parameter values and distributions is included in **Supplementary Table 3**. A summary of the parameters and source data is provided below.

Adenoma and SSL prevalence and features

Assumptions regarding precursors (prevalence, multiplicity, localization, size) were informed by our published systematic literature review.[6] For adenomas, the source data summarized in that review comprised autopsy series from before the introduction of screening (non-systematically reviewed), to avoid screening contamination;[10–20] for SSLs, the included source data comprised colonoscopy studies from a more recent era.[6] Although the SSL data may be subject to some screening contamination, observations were not significantly different for patients in any screening vs. first-time screening,[21] while more precise. Only European and North American studies were included for SSL estimates, given similar estimates of prevalence for those world regions but significant variation in other regions. To obtain estimates of prevalence from colonoscopy detection rates of SSLs, we accounted for a lesion miss rate of 25% observed in a meta-analysis of tandem colonoscopy studies (miss rate of 24-25% for serrated colorectal lesions).[22] Accounting for observed multiplicity, this lesion miss rate translated to a 20% patient miss rate. This patient miss rate was used as follows to convert detection to estimated prevalence in the natural history model: $Pr = Dr / (1-Mr)$. The miss rate was varied in sensitivity analyses.

For adenomas, data were stratified by age and sex, while for SSLs, only estimates by age or by sex were available.[6] To obtain adjusted age and sex-specific calibration targets, we scaled observed age-specific SSL data up/down by the square root of the relative ratio of prevalence by sex (ratio, 1.20).[6] Polyp multiplicity, localization, and size data were not age or sex-specific, and were assumed to represent the average across screening-eligible men or women (ages 50-74 years).

Cancer incidence

CRC incidence data by age, sex, and stage were derived from Surveillance Epidemiology and End Results (SEER) program data.[23] We used data from the 9 original geographic areas within SEER (San Francisco and Oakland, California; Connecticut; Detroit, Michigan; Hawaii; Iowa; New Mexico; Seattle and Puget Sound, Washington; Utah; and Atlanta, Georgia), from 1990-1994, before CRC screening was widespread. Stages were categorized as I, II, III and IV following the American Joint Committee on Cancer (AJCC) Screening's 3rd staging manual edition, which was checked for consistency with later editions defining additional substages. In sensitivity analysis, we evaluated increased incidence (IRR 1.54) considering observed trends in early-onset CRC incidence.[24,25]

Mortality

Colorectal cancer survival by age, sex, stage and location was estimated using SEER data from 2004-2015, with staging based on the AJCC's 6th cancer staging manual.[23] Cause-specific survival data are summarized in **Supplementary Figure 5**. After 10 years, we assumed a low rate of mortality from CRC equal to the average rate of the last three observed years, given most cancers are considered cured after 5-10 years but cancer may return. Location was categorized as proximal colon (cecum, ascending colon, hepatic flexure, transverse colon), distal colon (splenic flexure, descending colon, sigmoid colon), and rectum (incl. rectosigmoid junction). No survival differences were assumed for CRCs deriving from adenomas vs. SSLs, due to lack of high-quality data to inform those.

Competing risk of death was based on 2017 U.S. life tables.[26]

Tests

Test performance data were from a systematic review for colonoscopy,[22] a large population-based study for fecal immunochemical testing (cutoff of 20 µg stool/g stool; FIT-20) and the current stool-DNA/FIT (sDNA-FIT),[27] a systematic review on FIT with a cutoff of 10 µg/g (FIT-10),[28] and a verification study of a new sDNA-FIT (sDNA-FIT2.0) (**Table 1** of the article).[29] These studies did not consistently report the sensitivity for all lesion types that we distinguished in the model; therefore, some assumptions had to be made.

First, for colonoscopy, we used performance estimates from studies of conventional colonoscopy followed by enhanced colonoscopy (Design A in Zhao et al). Zhao et al. provided estimates of size/stage-specific adenoma sensitivity, but not size/stage-specific SSL sensitivity.[22] Since estimates of overall adenoma and SSL miss-rates were similar, we assumed equal sensitivity for adenomas and SSLs by size and stage; lower SSL sensitivity was evaluated in sensitivity analysis. Sensitivity for CRCs was not reported and assumed similar to large adenomas.

For noninvasive tests, we assumed reported performance against colonoscopy to be representative of actual test performance. For FIT-20 and sDNA-FIT, Imperiale et al. reported adenoma sensitivity for nonadvanced (incl. small-medium tubular) *vs*. advanced adenomas (incl. large), and for large SSLs.[27] Since the reported sensitivity of FIT-20 for large SSLs was similar to its lack of specificity, we assumed no sensitivity of FIT-20 for SSLs of any size. Since the reported sensitivity of sDNA-FIT for large adenomas and SSLs was similar, we assumed the same sensitivity for small or medium adenomas and SSLs for sDNA-FIT. The systematic review by Imperiale et al. and the pilot study by Kisiel et al. did not report estimates of SSL sensitivity altogether.[28,29] We made similar assumptions for FIT-10 vs FIT-20 regarding the lack of sensitivity for SSLs, and for sDNA-FIT vs sDNA-FIT2.0 regarding equal sensitivity for adenomas vs SSLs.

Colonoscopy complication rates were based on a systematic review commissioned for the 2021 US Preventive Services Task Force CRC screening recommendation,[30] distinguishing adverse bleeding events and perforations.

Recurrence of lesions

Presence of adenomas and SSLs three-five years after polypectomy was informed by eight pooled North American studies,[31] and data from the New Hampshire Colonoscopy Registry,[32] respectively (details in **Model calibration**). Detection of advanced lesions, especially, at surveillance may be attributable to missed lesions at prior colonoscopy, incomplete removal of lesions, or rapid development of new lesions.[33] The corresponding attributable fractions are unknown. We applied excess advanced-lesion recurrence after polypectomy on top of polyp miss rates and normal background incidence to match observed detection of advanced lesions in surveillance, without specifying whether this was due to residual tissue or new lesions.

Costs

CRC-related procedure costs including geographic variation were derived from 2020 U.S. Centers for Medicare and Medicaid Services (CMS) data.[34] For FIT and sDNA-FIT we used the Clinical Laboratory Fee Schedule to approximate cost. For colonoscopy and colonoscopy-related complications, we used relevant combinations in-office, out-patient, ambulatory and in-patient reimbursement rates from the National Physician Fee Schedule.

CMS reimbursement rates vary between counties. To account for this, we varied rates according to CMS' Geographic Variation Public Use File.[35] We obtained case-neutral geographic adjustment factors by dividing geography-adjusted payments by raw payments for each county (**Supplementary Figure 6**), and normalizing the result to have unit mean. We then applied these case-adjusted, normalized factors against national reimbursement rates to obtain distributions of costs, applying test factors for FIT and sDNA-FIT costs, procedure factors for colonoscopy costs, and in-patient factors for complications (more detail under **Uncertainty analysis**).

Annualized CRC treatment costs by stage and care phase were derived from a SEER-CMS linkage analysis,[36] indexed to 2020 using the U.S. consumer price index.[37] By comparing costs between CRC patients and matched controls, Mariotto

et al. derived net CRC cost estimates. Costs distinguished the four AJCC stages and three care phases: initial, continuing, and terminal care (death from CRC or other causes). Initial care was defined as the period up to 12 months after diagnosis, in case of over 12 months until death; terminal care as the last 12 months of CRC care before death; and continuing care as the period in between. In the model, we assumed no costs of treatment during the continuing phase after 5 years from diagnosis.

Utilities

Disutility or quality-of-life estimates were based on estimated duration and burden of procedures (similar to other models),[38] and a standard-gamble study reflecting patients' treatment preferences.[39] We assumed a four hour loss in quality of life for FIT and sDNA-FIT reflecting the worry awaiting test results and follow-up, a 16 hour loss in quality of life for colonoscopy, 48 hours or two days for adverse bleeding following colonoscopy, and 168 hours or seven days for perforation. Stage-specific losses in quality of life associated with CRC treatment were based on patient preferences elicited by Ness et al.[39] regarding different forms of treatment. No loss in quality of life was assumed in the continuing phase after five years of treatment.

Model calibration

Some model features are directly informed by observed data; others require calibration. Calibration is needed when no direct observations on an assumption exist, but when related outcome data are informative; calibration searches the model parameter values that best reproduce those outcomes.

In ANSER, calibration was required for assumptions spanning the CRC natural history in the model. In total, these assumptions were calibrated in six conditionally independent steps: 1) initial adenoma and SSL formation; 2) subsequent lesion formation after the first lesion; 3) lesion growth; 4) CRC stage progression and symptom development; 5) progression of precursors to CRC; 6) lesion recurrence after removal.

Steps 1)-3) and 5) used a search strategy in between random search and exhaustive grid search: Latin hypercube sampling, to efficiently roam the space of possible parameter values;[40,41] the other two steps used algorithmic *vs*. manual calibration, respectively, given the high-dimensionality and distinct model stage (post-screening). Model fit was evaluated using log-likelihood criteria, aggregated by age, sex, lesion number, size, or stage, as applicable.[42] We initially cast a wide net in terms of parameter values considered, narrowing this down to smaller ranges evaluated at a higher resolution of sampled parameter values, to then retain the set of best-fitting model parameter values consistent with 95% confidence intervals (95%CIs) around the observed data, as available. Subsequently, we permutated these values with newly sampled parameter values relevant to the next outcome, and repeated the above steps. In the end, we retained 100 parameter sets reflective of uncertainty in the source data (see **Uncertainty analysis**). Evidence of successful calibration is provided in **Supplementary Figures 2-4**. More details on each of the steps are provided below.

Precursor onset and growth

First, we calibrated the rate of initial precursor lesion formation (6 parameters) to observed adenoma (117 data points) and SSL prevalence (20 data points) by age and sex across studies in our systematic review (**Supplementary Table 1**).[6] Two time-to-event distributions were considered for reproducing the observed data: a Gompertz distribution and a Weibull distribution, each defined by calibrated shape and scale parameters. Of the two, the Weibull distribution provided the best goodness of fit. Without 95%CIs available for the scatter of observed data points by age, we used our subjective judgment to select the number of best-fitting assumptions reflective of uncertainty (different lines in **Supplementary Figure 2**).

Second, we calibrated subsequent lesion formation parameters (4 for each sex) to the observed multiplicities in our systematic review (6 data points) (**Supplementary Table 1**), assuming no CRC progression at this point to ensure identifiability.[6] We evaluated an exponential time-to-event distribution, with fixed incidence over time. We retained calibrated rate parameters consistent with the 95%CIs around the meta-estimates of the proportions with one, two or three+ lesions (**Supplementary Figure**

3). While each proportion was estimated separately in meta-analysis, with differential standard errors and 95%CIs resulting, proportions in the model should add up to 100%. Respecting this dependence, we anchored our calibration to the most precisely estimated proportions in meta-analysis recognizing that this may underestimate uncertainty.

Third, adenoma and SSL growth assumptions (4 parameters) were calibrated to the respective size distributions (6 data targets) in our systematic review (**Supplementary Table 1**),[6] in a similar fashion. We evaluated fixed annual transition rates for approximate exponential time to growth (in a Markov-like process), and we retained those rate parameters consistent with the 95%CIs around meta-estimates of the population proportions with 0-5mm, 6-9mm or 10+mm adenomas or SSLs (**Supplementary Figure 3**).

Cancer incidence and stage

Rates of CRC stage progression (24 parameters) and symptom development (16 parameters) by age and stage were calibrated to SEER incidence by age and stage (56 data points) (**Supplementary Table 1**).[23] Considering the high dimensionality of this optimization problem, we first utilized a Generalized Reduced Gradient algorithm to find 11 CRC stage-related parameter sets consistent with CRC sojourn times of 3-5 years (0.2 year increments), i.e. the plausible range from literature and other models.[43–45] We tried different starting values for optimization with similar resulting parameters. These parameters were permuted with adenoma and SSL progression assumptions in the following step, before selecting optimal parameters for CRC incidence by age, sex and stage.

In that following step, we calibrated rates of progression of advanced (large) adenomas and SSLs by age and sex (8 parameters) to observed overall CRC incidence by age, stage and sex (112 data points) (**Supplementary Table 1**).[23] The calibration routine for incidence was similar as for other natural history assumptions, using iterative Latin hypercube sampling. We focused on the most relevant age range of 40-80 years. Observed decreasing incidence after age 80 years was considered an artifact of underreporting rather than biology, and not picked up by the

model. In the end, we selected the 100 best-fitting parameter sets consistent with 95%CIs around observed rates (**Supplementary Figure 4**).

Post-polypectomy lesion recurrence

At a later stage in model development, we calibrated recurrence rates of advanced adenomas and SSLs (6 parameters) after polypectomy to observed detection rates in surveillance (6 data points).[31,32] Unlike in other calibration steps, we did not use computer-assisted search but manually adjusted recurrence rates of advanced lesions and of CRC, to best match observed rates across different model assumptions. No increased recurrence of nonadvanced lesions was assumed on top of normal background incidence since the higher colonoscopy miss rates for those lesions and the standard onset of new lesions combined generated sufficient nonadvanced lesions in surveillance to match observed detection rates.

Identifiability

In designing our model, we minimized the number of model parameters necessary to reproduce the observed data targets. Most model assumptions were independently identifiable conditional on prior calibration steps, given the assumed one-directional nature of carcinogenesis and the ratio of calibrated parameters to observed data points (**Supplementary Table 1**). In Step 4), source data were not informative on whether cancers diagnosed in stage II spent more time in preclinical stage I or preclinical stage II, et cetera. We did not propagate this uncertainty, but only used the best-fitting sets of parameters for each sojourn time considered (with calibrated time in stage I turning out somewhat longer than the times in other stages).

Model uncertainty

Uncertainty in model outcomes was estimated using an approach similar to that described by Weinstein and others.[46–49] We sampled plausible sets of model parameter values based on calibration and direct data sources, ran the model for each set, and derived *95% uncertainty intervals* (95%UIs) across resulting

outcomes, which we defined as the 2.5-97.5 percentile range. Effectively, this assumes all evaluated parameter sets to be equally probable.

Natural history parameter values were bootstrapped from the family of parameter sets obtained through calibration. Test, cost and disutility parameter values were sampled from beta or lognormal distributions fitted to observed means and 95%CIs, to the extent available. Different approaches were needed for test - and complication costs / disutility: for costs, we used geographic variation as our best approximation of the uncertainty in costs underlying otherwise fixed reimbursements; for disutility, we applied agnostic +/- 50% uniform distributions in the absence of data to inform these assumptions, in light of contradictory expert recommendations against both arbitrary ranges and exclusion of parameters lacking informative data.[49] Considering the difficulty of estimating utility, the selected range best reflects our own confidence in these estimates. Mortality rates were not varied due to the relatively high precision of mortality rates.

Correlation between natural history parameters was derived directly from the calibrated families of parameters sets. For test performance assumptions, we assumed: correlation of FIT and sDNA-FIT sensitivities for adenomas and CRC, given the similar mechanisms for detection; independence of sensitivity for adenomas and SSLs, given FIT's complete lack of sensitivity for SSLs; and, inverse correlation of test sensitivities and specificities. For costs and disutility, we assumed correlation within all early treatment phases; within advanced treatment phases; between colonoscopy with and without polypectomy; and between the main colonoscopy complications.

Cross-model validation

To assess differences in natural history vs. other models, we compared the model's dwell times for precursor lesions until progression to preclinical CRC, the sojourn times for preclinical CRCs until clinical diagnosis, 20-year CRC incidence from age 55 years for individuals with vs. without such lesions, and, the 15-year incidence reduction for a perfectly sensitive exam at age 65 years (maximum clinical incidence reduction, MCLIR) with previously published estimates by the Cancer Intervention

and Surveillance Modeling Network (CISNET). With base-case assumptions and the best-fitting set of model parameters, mean dwell time in ANSER was 27.6 years for SSLs (IQR, 19-35) and 29.0 years for adenomas (IQR, 20-37) vs. a mean 7.6-24.2 years for three CISNET models;[50] CRC sojourn time was 3.2 years (IQR, 2-4) vs. 1.6-4.0 years for three CISNET models;[50] 20-year incidence from age 55 years was <0.1% for persons without lesions or preclinical cancer (similar for three CISNET models) compared to 8.7% for persons with lesions (approximately 8-15% for three CISNET models);[50] and the MCLIR was 49.8% vs. 51-90% for three CISNET models.[51] The relatively longer dwell times and lower risk in ANSER could be explained by the addition of serrated precursor lesions (more lesions, similar incidence) and the lower CRC rates for 1990-1994 that we used for calibration vs. the 1975-1979 rates used by CISNET. The lower MCLIR in ANSER was due to assumed excess advanced lesion recurrence after lesion removal (e.g. due to inadequate resection); the MCLIR increased to 69.8% when this possibility of excess advanced-lesion recurrence was silenced in the model. Overall, these natural history features are consistent with the range reflected in the three CISNET models.

To evaluate convergence in outcomes, we also compared predictions from ANSER for several common screening strategies with those from three other well-established models, including the most and least conservative CISNET models and an often-cited Markov model.[25,38,52,53] For the best-fitting set of natural history parameters, predictions from ANSER were largely in the range of other models (**Supplementary Table 3**). ANSER's predicted incidence and mortality were lower compared to the two CISNET models, which spilled over into other outcomes.[38,52] Again, this difference reflects the CRC rates during 1990-1994 *vs*.1975-1979, the respective calibration periods for background incidence. Compared to the Markov model, which used the same period incidence as ANSER,[25] predictions were closely aligned. When we assumed higher background incidence to incorporate trends in early-onset incidence, with incidence rate ratios (IRRs) of 1.54, ANSERs predictions were also close to those from the two CISNET models.[53] Colonoscopy screening costs were consistently lower in ANSER than in other models, partly due to our implementation of the current, less intensive U.S. surveillance guidelines for patients with removed lower-risk adenomas,[54] and a health-sector perspective.[55]

External model validation

Annual stool-based screening outcomes were validated against the Minnesota Colorectal Cancer Control Study after 13-18 years follow-up.[56] To replicate the study, we simulated a cohort of 63-year old adults, consisting of 52% women and 48% men. Screening comprised 5 annual rounds of guaiac-based fecal occult blood testing (FOBT), followed by 5 years without screening, and another 6 years of annual testing. Participants with a positive FOBT result were referred for follow-up colonoscopy. Participant adherence to FOBT and follow-up colonoscopy were based on the reported rates in the trial: 46% completed all FOBTs, 44% completed 1-99% of all tests, 10% completed none of the FOBTs, and 83% completed follow-up colonoscopy after a positive FOBT result. Diagnostic performance of FOBT for CRC was also based on the trial data (92.2% for predominant rehydrated FOBT),[57] but sensitivity for precursor lesions was as assumed in model analyses for the US Preventive Services Task Force (7.5-23.9%).[52,53] Diagnostic performance of colonoscopy was as described in **Table 1** of the article. We calculated cumulative incidence of CRC cases and deaths over a period of 18 years follow-up. The effect of screening was derived as the ratio of the cumulative incidence and mortality over this period for the screening *vs*. control arm, weighted by person-years of follow-up in the trial.

Endoscopic screening outcomes were validated against *per-protocol* results from the UK Flexible Sigmoidoscopy Study after 11 years follow-up.[58] To replicate this study, we simulated a cohort of 60-year old adults, 51% women and 49% men. Screening consisted of a single flexible sigmoidoscopy (FS) at baseline. Similar to the trial protocol, patients with ≥3 adenomas or ≥1 large adenoma were referred for colonoscopy follow-up. Participant adherence to FS and follow-up colonoscopy was based on the reported rates in the trial: 71% completed FS, and 96% completed follow-up colonoscopy (only adherent patients were considered in per-protocol analysis). Diagnostic accuracy for FS was assumed to be similar to colonoscopy (**Table 1** of the article), but was assumed to reach no further than the distal colon and rectum, and to have 50% reduced sensitivity for SSLs compared to adenomas given SSLs were less well understood when this study was performed. We calculated cumulative incidence of CRC cases and deaths over a period of 11 years follow-up, as the complement of Kaplan-Meier estimates of disease-free survival.

The effect of screening was summarized as the ratio of the average cumulative incidence and mortality over this period for the screening *vs*. control arm, weighted by the person-years of follow-up in the trial.

Validation results are summarized in **Figure 2** and discussed in the article.

References

1. R Core Team (2022). R: A language and environment for statistical computing. R Foundation for Statistical Computing, Vienna, Austria. https://www.R-project.org/.

2. A. M. Law and W. D. Kelton. Simulation Modeling and Analysis, 3rd Edition, McGraw-Hill Inc., New York, 2000.

3. Muto T, Bussey HJ, Morson BC. The evolution of cancer of the colon and rectum. Cancer 1975;36(6):2251–70.

4. O'Brien MJ, Winawer SJ, Zauber AG, et al. The National Polyp Study. Patient and polyp characteristics associated with high-grade dysplasia in colorectal adenomas. Gastroenterology 1990;98(2):371–9.

5. Turner KO, Genta RM, Sonnenberg A. Lesions of All Types Exist in Colon Polyps of All Sizes. Am J Gastroenterol 2018;113(2):303–6.

6. Meester RGS, van Herk MMAGC, Lansdorp-Vogelaar I, Ladabaum U. Prevalence and Clinical Features of Sessile Serrated Polyps: A Systematic Review. Gastroenterology 2020;159(1):105-118.e25.

7. Digestive System Tumours. WHO Classification of Tumours, 5th Edition. International Agency for Cancer Research;

8. Anderson JC, Butterly LF, Weiss JE, Robinson CM. Providing data for serrated polyp detection rate benchmarks: an analysis of the New Hampshire Colonoscopy Registry. Gastrointest Endosc 2017;85(6):1188–94.

9. Pérez-Martín J, Bermejo I, Díez FJ. Evaluation of Markov Models with Discontinuities. Med Decis Making 2019;39(4):414–20.

10. Arminski TC, Mclean DW. Incidence and distribution of adenomatous polyps of the colon and rectum based on 1,000 autopsy examinations. Dis Colon Rectum 1964;7:249–61.

11. Blatt L. Polyps of the colon and rectum: incidence and distribution. Dis Colon Rectum 1961;(4):277–82.

12. Bombi JA. Polyps of the colon in Barcelona, Spain. An autopsy study. Cancer 1988;61(7):1472–6.

13. Chapman I. Adenomatous polypi of large intestine: incidence and distribution. Ann Surg 1963;157(2):223–6.

14. Clark JC, Collan Y, Eide TJ, et al. Prevalence of polyps in an autopsy series from areas with varying incidence of large-bowel cancer. Int J Cancer 1985;36(2):179–86.

15. Eide TJ, Stalsberg H. Polyps of the large intestine in Northern Norway. Cancer 1978;42(6):2839–48.

16. Jass JR, Young PJ, Robinson EM. Predictors of presence, multiplicity, size and dysplasia of colorectal adenomas. A necropsy study in New Zealand. Gut 1992;33(11):1508–14.

17. Rickert RR, Auerbach O, Garfinkel L, Hammond EC, Frasca JM. Adenomatous lesions of the large bowel: an autopsy survey. Cancer 1979;43(5):1847–57.

18. Stemmermann GN, Yatani R. Diverticulosis and polyps of the large intestine. A necropsy study of Hawaii Japanese. Cancer 1973;31(5):1260–70.

19. Vatn MH, Stalsberg H. The prevalence of polyps of the large intestine in Oslo: an autopsy study. Cancer 1982;49(4):819–25.

20. Williams AR, Balasooriya BA, Day DW. Polyps and cancer of the large bowel: a necropsy study in Liverpool. Gut 1982;23(10):835–42.

21. Li D, Woolfrey J, Jiang S-F, et al. Diagnosis and predictors of sessile serrated adenoma after educational training in a large, community-based, integrated healthcare setting. Gastrointest Endosc 2018;87(3):755-765.e1.

22. Zhao S, Wang S, Pan P, et al. Magnitude, Risk Factors, and Factors Associated With Adenoma Miss Rate of Tandem Colonoscopy: A Systematic Review and Meta-analysis. Gastroenterology 2019;156(6):1661-1674.e11.

23. SEER*Stat Database: Incidence—SEER 9 Regs Research Data with Delay-Adjustment, Malignant Only, Nov 2018 Sub (1975-2016) <Katrina/Rita Population Adjustment>—Linked To County Attributes—Total US, 1969-2017 Counties. National Cancer Institute. Released April 2019.

24. Siegel RL, Fedewa SA, Anderson WF, et al. Colorectal Cancer Incidence Patterns in the United States, 1974-2013. J Natl Cancer Inst 2017;109(8):djw322.

25. Ladabaum U, Mannalithara A, Meester RGS, Gupta S, Schoen RE. Cost-Effectiveness and National Effects of Initiating Colorectal Cancer Screening for Average-Risk Persons at Age 45 Years Instead of 50 Years. Gastroenterology 2019;157(1):137–48.

26. Arias E. United States Life Tables, 2017. Natl Vital Stat Rep 2019;68(7):1–66.

27. Imperiale TF, Ransohoff DF, Itzkowitz SH, et al. Multitarget stool DNA testing for colorectal-cancer screening. N Engl J Med 2014;370(14):1287–97.

28. Imperiale TF, Gruber RN, Stump TE, Emmett TW, Monahan PO. Performance Characteristics of Fecal Immunochemical Tests for Colorectal Cancer and Advanced Adenomatous Polyps: A Systematic Review and Meta-analysis. Ann Intern Med 2019;170(5):319–29.

29. Kisiel JB, Gagrat ZD, Krockenberger M, et al. Can second-generation multitarget stool DNA panels reliably detect colorectal cancer and advanced precancerous lesions? JCO 2022;40(4_suppl):63–63.

30. Lin JS, Perdue LA, Henrikson NB, Bean SI, Blasi PR. Screening for Colorectal Cancer: Updated Evidence Report and Systematic Review for the US Preventive Services Task Force. JAMA 2021;325(19):1978–98.

31. Martínez ME, Baron JA, Lieberman DA, et al. A pooled analysis of advanced colorectal neoplasia diagnoses after colonoscopic polypectomy. Gastroenterology 2009;136(3):832–41.

32. Anderson JC, Butterly LF, Robinson CM, Weiss JE, Amos C, Srivastava A. Risk of Metachronous High-Risk Adenomas and Large Serrated Polyps in Individuals With Serrated Polyps on

Index Colonoscopy: Data From the New Hampshire Colonoscopy Registry. Gastroenterology 2018;154(1):117-127.e2.

33. Pohl H, Anderson JC, Aguilera-Fish A, Calderwood AH, Mackenzie TA, Robertson DJ. Recurrence of Colorectal Neoplastic Polyps After Incomplete Resection. Ann Intern Med 2021;174(10):1377–84.

34. US Centers for Medicare and Medicaid Services. CMS Fee Schedules - General Information [Internet]. [cited 2023 Jun 23];Available from: https://www.cms.gov/medicare/medicare-fee-for-service-payment/feeschedulegeninfo

35. US Centers for Medicare and Medicaid Services. Medicare Geographic Variation - by National, State & County [Internet]. [cited 2022 Jul 1];Available from: data.cms.gov

36. Mariotto AB, Warren JL, Zeruto C, et al. Cancer-Attributable Medical Costs for Colorectal Cancer Patients by Phases of Care: What Is the Effect of a Prior Cancer History? J Natl Cancer Inst Monogr 2020;2020(55):22–30.

37. Bureau of Labor Statistics Data [Internet]. [cited 2023 Jun 23];Available from: https://data.bls.gov/cgi-bin/surveymost

38. Peterse EFP, Meester RGS, de Jonge L, et al. Comparing the Cost-Effectiveness of Innovative Colorectal Cancer Screening Tests. J Natl Cancer Inst 2021;113(2):154–61.

39. Ness RM, Holmes AM, Klein R, Dittus R. Utility valuations for outcome states of colorectal cancer. Am J Gastroenterol 1999;94(6):1650–7.

40. McKay MD, Beckman RJ, Conover WJ. A Comparison of Three Methods for Selecting Values of Input Variables in the Analysis of Output from a Computer Code. Technometrics 1979;21(2):239–45.

41. Stout NK, Knudsen AB, Kong CY, McMahon PM, Gazelle GS. Calibration methods used in cancer simulation models and suggested reporting guidelines. Pharmacoeconomics 2009;27(7):533–45.

42. van der Steen A, van Rosmalen J, Kroep S, et al. Calibrating Parameters for Microsimulation Disease Models: A Review and Comparison of Different Goodness-of-Fit Criteria. Med Decis Making 2016;36(5):652–65.

43. Zheng W, Rutter CM. Estimated mean sojourn time associated with hemoccult SENSA for detection of proximal and distal colorectal cancer. Cancer Epidemiol Biomarkers Prev 2012;21(10):1722–30.

44. Brenner H, Altenhofen L, Katalinic A, Lansdorp-Vogelaar I, Hoffmeister M. Sojourn time of preclinical colorectal cancer by sex and age: estimates from the German national screening colonoscopy database. Am J Epidemiol 2011;174(10):1140–6.

45. Rutter CM, Knudsen AB, Marsh TL, et al. Validation of Models Used to Inform Colorectal Cancer Screening Guidelines: Accuracy and Implications. Med Decis Making 2016;36(5):604–14.

46. Weinstein MC. Recent developments in decision-analytic modelling for economic evaluation. Pharmacoeconomics 2006;24(11):1043–53.

47. Kim JJ, Kuntz KM, Stout NK, et al. Multiparameter calibration of a natural history model of cervical cancer. Am J Epidemiol 2007;166(2):137–50.

48. Taylor DCA, Pawar V, Kruzikas D, et al. Methods of model calibration: observations from a mathematical model of cervical cancer. Pharmacoeconomics 2010;28(11):995–1000.

49. Briggs AH, Weinstein MC, Fenwick EAL, et al. Model parameter estimation and uncertainty: a report of the ISPOR-SMDM Modeling Good Research Practices Task Force--6. Value Health 2012;15(6):835–42.

50. Kuntz KM, Lansdorp-Vogelaar I, Rutter CM, et al. A systematic comparison of microsimulation models of colorectal cancer: the role of assumptions about adenoma progression. Med Decis Making 2011;31(4):530–9.

51. van Ballegooijen M, Rutter CM, Knudsen AB, et al. Clarifying differences in natural history between models of screening: the case of colorectal cancer. Med Decis Making 2011;31(4):540–9.

52. Knudsen AB, Zauber AG, Rutter CM, et al. Estimation of Benefits, Burden, and Harms of Colorectal Cancer Screening Strategies: Modeling Study for the US Preventive Services Task Force. JAMA 2016;315(23):2595–609.

53. Knudsen AB, Rutter CM, Peterse EFP, et al. Colorectal Cancer Screening: An Updated Modeling Study for the US Preventive Services Task Force. JAMA 2021;325(19):1998–2011.

54. Gupta S, Lieberman D, Anderson JC, et al. Recommendations for Follow-Up After Colonoscopy and Polypectomy: A Consensus Update by the US Multi-Society Task Force on Colorectal Cancer. Gastroenterology 2020;158(4):1131-1153.e5.

55. Sanders GD, Neumann PJ, Basu A, et al. Recommendations for Conduct, Methodological Practices, and Reporting of Cost-effectiveness Analyses: Second Panel on Cost-Effectiveness in Health and Medicine. JAMA 2016;316(10):1093–103.

56. Mandel JS, Bond JH, Church TR, et al. Reducing mortality from colorectal cancer by screening for fecal occult blood. Minnesota Colon Cancer Control Study. N Engl J Med 1993;328(19):1365–71.

57. Church TR, Ederer F, Mandel JS. Fecal occult blood screening in the Minnesota study: sensitivity of the screening test. J Natl Cancer Inst 1997;89(19):1440–8.

58. Atkin WS, Edwards R, Kralj-Hans I, et al. Once-only flexible sigmoidoscopy screening in prevention of colorectal cancer: a multicentre randomised controlled trial. Lancet 2010;375(9726):1624–33.

Supplementary Figure 1. Natural history of colorectal cancer as simulated by ANSER.

In each simulated individual, CRC can develop from adenomas or sessile serrated lesions SSLs. ANSER can simulate multiple lesions per individual and distinguishes three sizes: diminutive (0-5mm), small (6-9mm), and large (≥10mm). When a large precursor lesion progresses to CRC, it may progress through stages I-IV before developing clinical symptoms. Clinically diagnosed CRC can result in early death, but individuals can also die from competing causes in any model state.

SSL denotes sessile serrated lesion, CRC colorectal cancer.

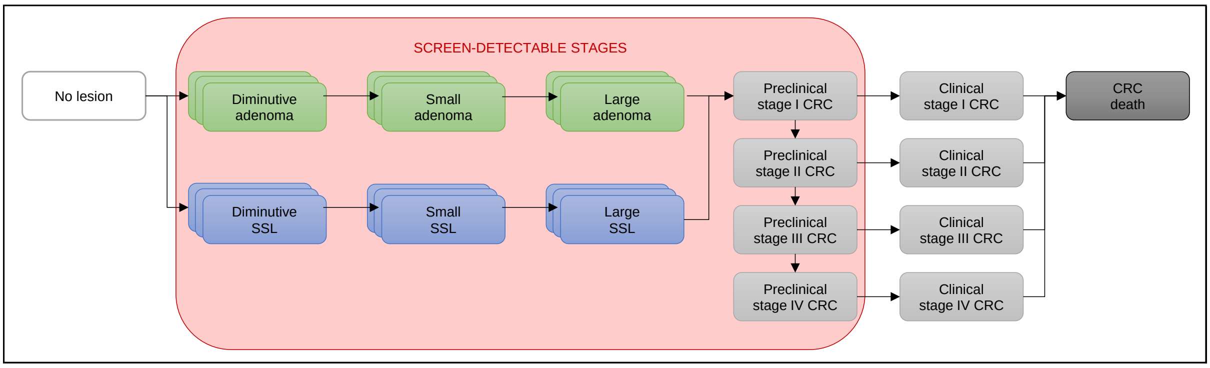

Supplementary Figure 2. Calibrated precursor prevalence by age and sex.

Dots represent published estimates of the prevalence of colorectal precancerous lesions;[6] lines represent the set of best-matching model versions. Sessile serrated lesions and traditional serrated adenomas were included as serrated polyps.

Abbreviations M = male; F=female.

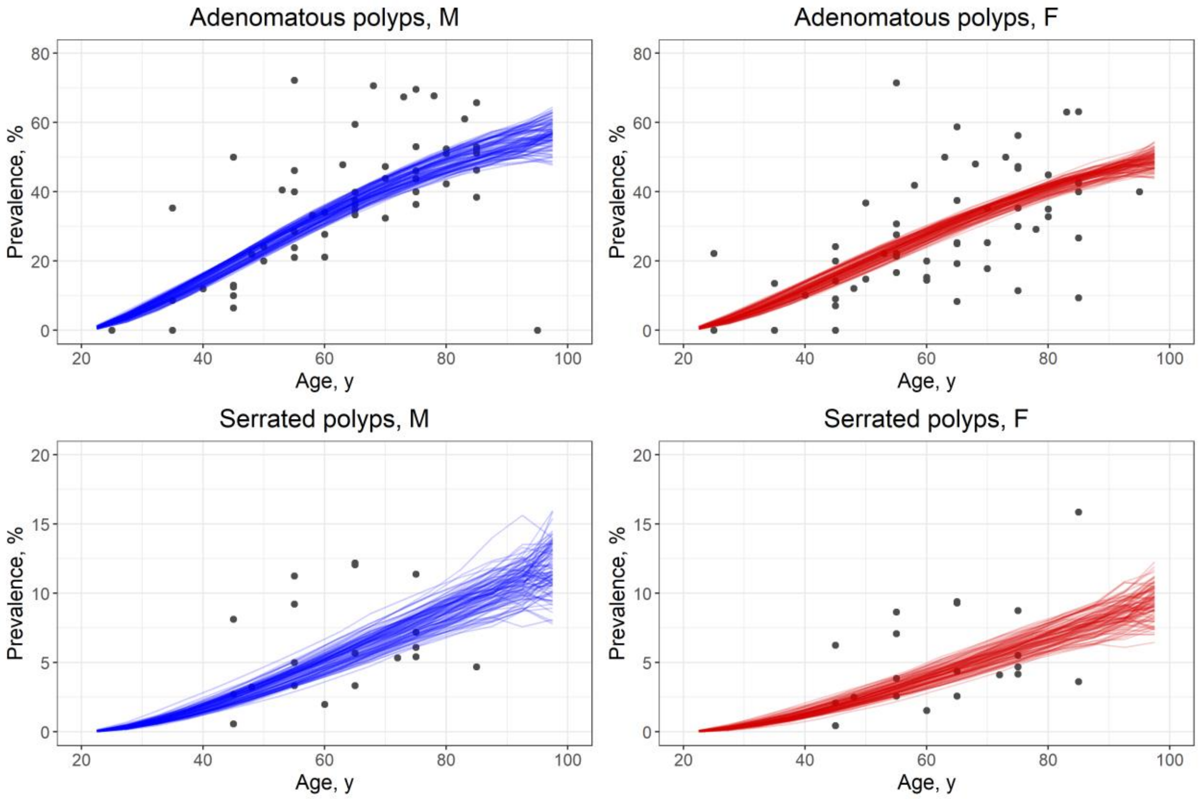

Supplementary Figure 3. Calibrated precursor features.

Bars represent meta-estimates of published literature;[6] whiskers represent 95% CIs varying in proportion to heterogeneity in published estimates; dots represent the set of best-matching model versions, calibrated to the narrowest 95% CI in each panel. Sessile serrated lesions and traditional serrated adenomas were included as serrated lesions.

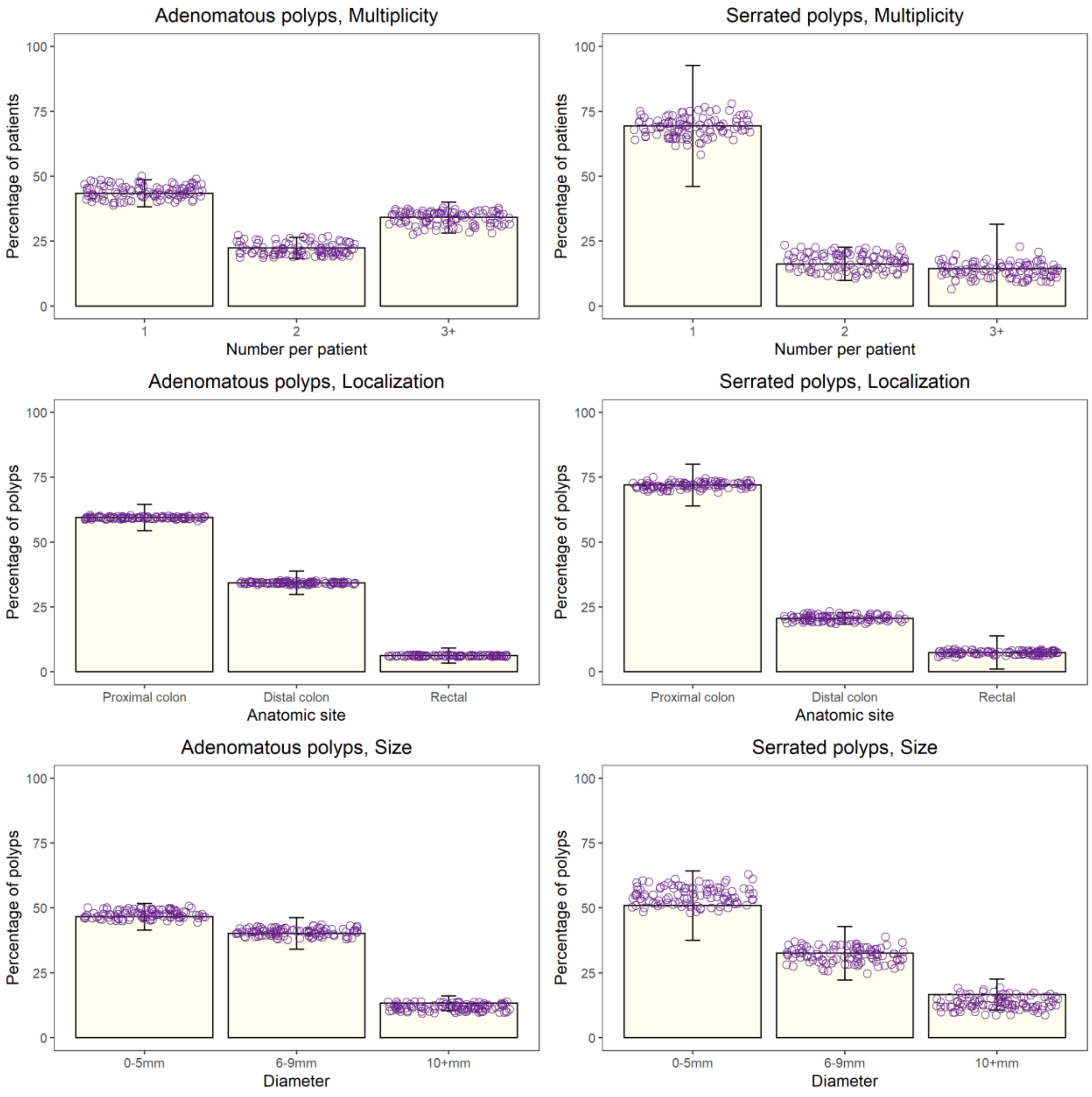

Supplementary Figure 4. Calibrated CRC incidence by age, sex and stage.

Dots represent observed estimates of colorectal cancer incidence in 1990-1994 Surveillance Epidemiology and End Results program data;[23] whiskers represent 95% CIs; lines represent the set of best-matching model versions. Stages were defined according to the 3rd edition of the American Joint Committee on Cancer Staging manual but consistent with the 8th edition.

M denotes male; F female.

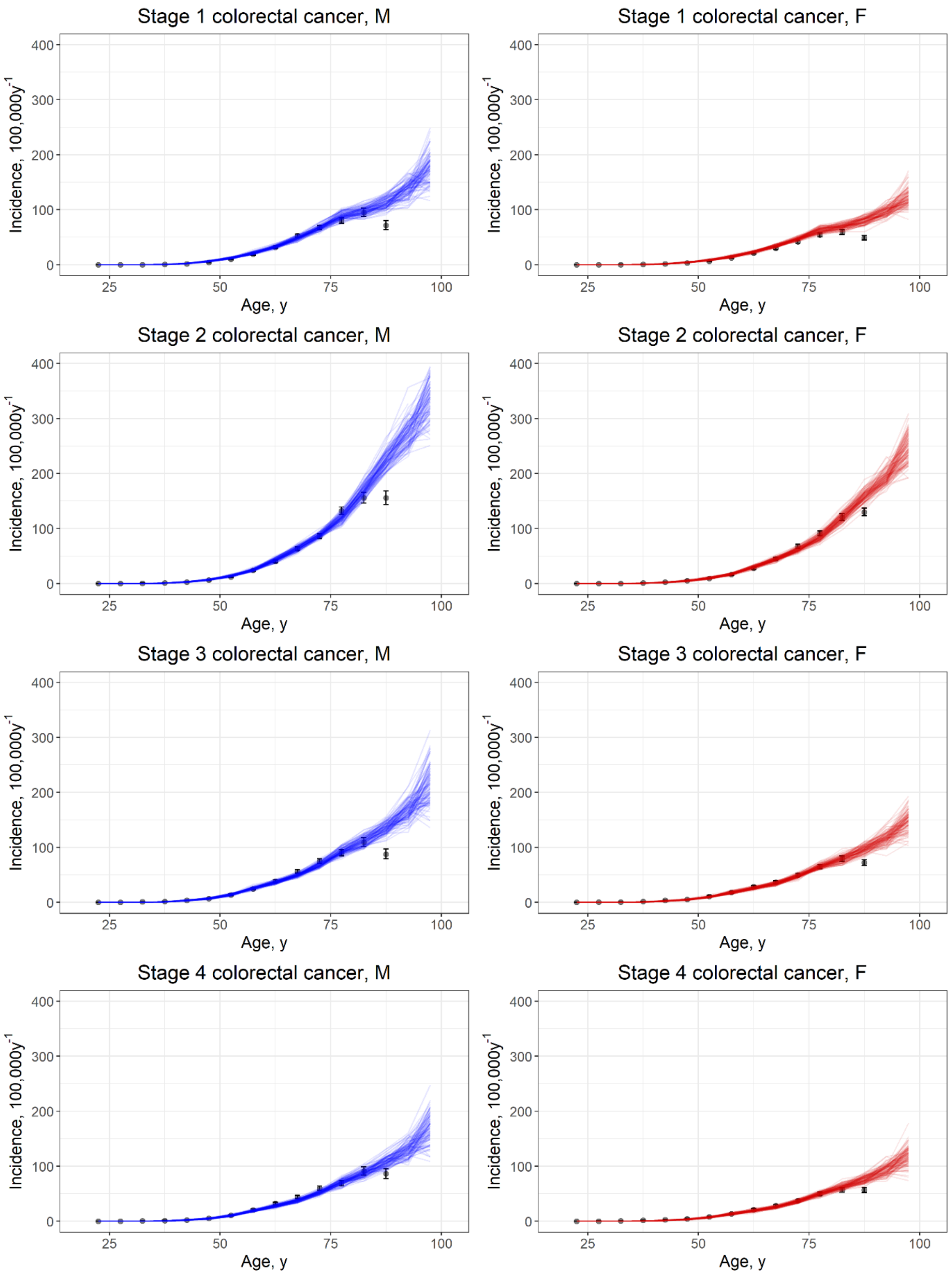

Supplementary Figure 5a. Observed CRC survival by age, stage and location in women.

Data were from cases diagnosed during 2004-2015 recorded in SEER.[23] Lines represent means, ribbons represent 95%CIs. In ANSER, mortality was assumed constant after year ten.

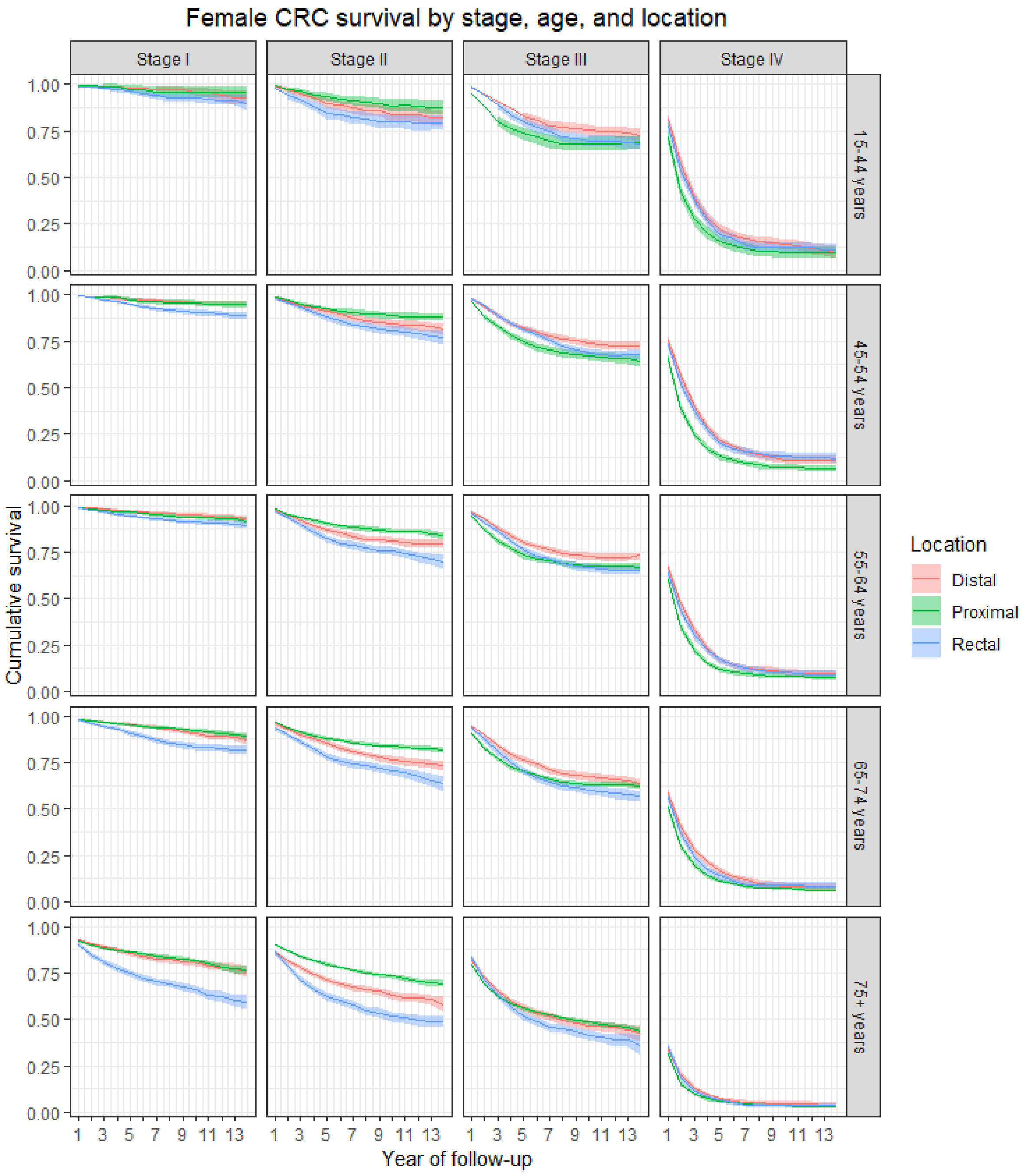

Supplementary Figure 5b. Observed CRC survival by age, stage and location in men.

Data were obtained from nine SEER during 2004-2015.[23] In ANSER, mortality was assumed constant after year ten.

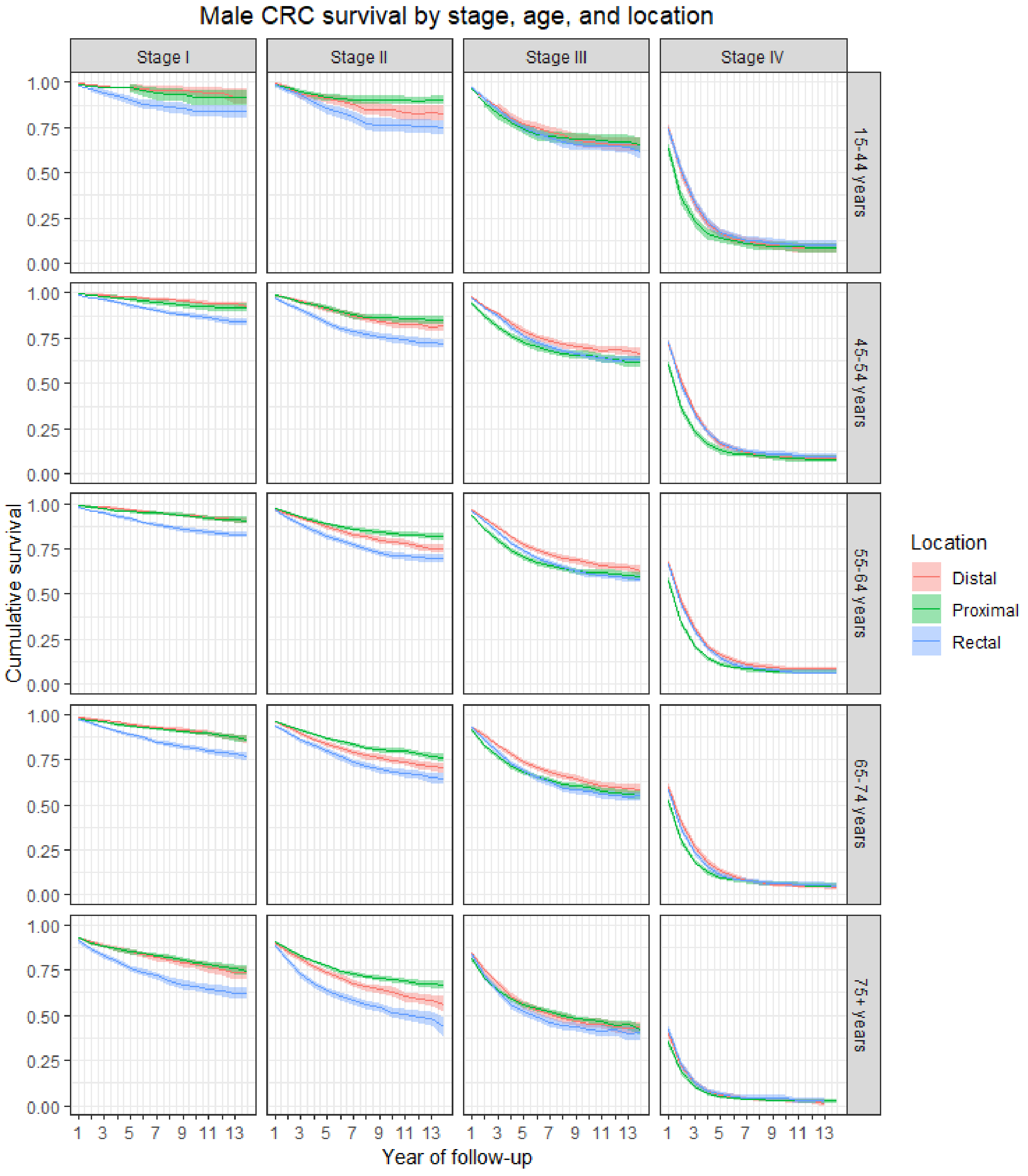

Supplementary Figure 6. Variation in CRC-related treatment, procedures, and tests.

Assumed cost variation was based on observed county-level variation in CMS reimbursements, adjusted for case-mix.[35] Raw geographic adjustment factors were normalized to vary around a mean of 1.

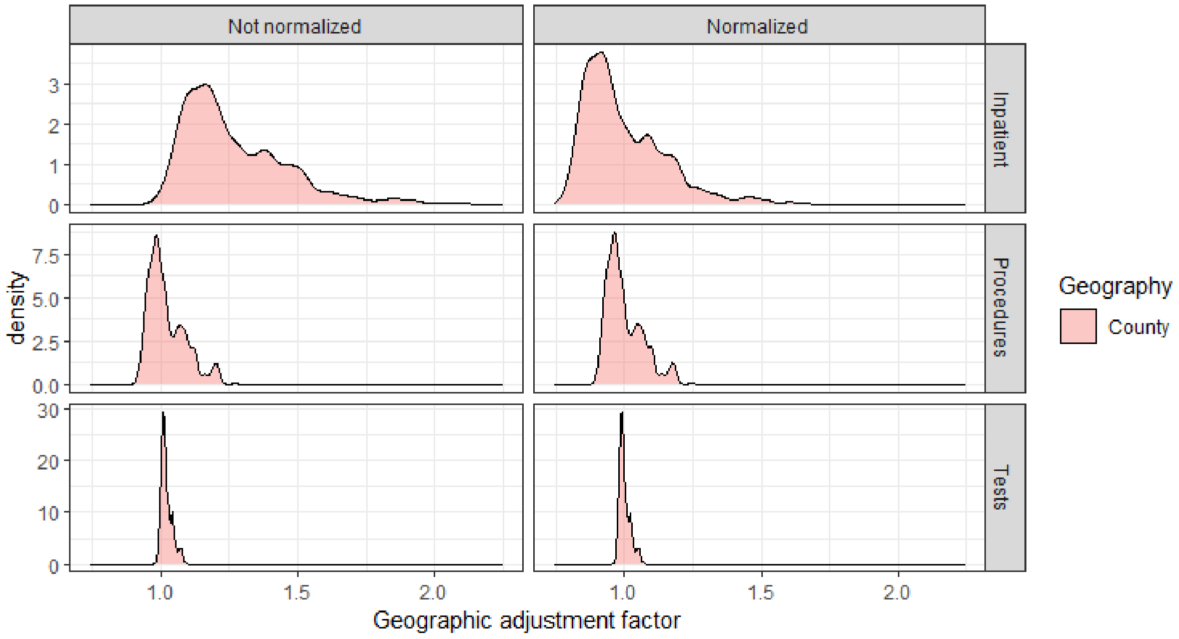

Supplementary Figure 7. Lifetime effectiveness, cost-effectiveness and burden-benefit of all strategies.

Figures compare selected screening strategies in terms of simulated lifetime colorectal cancer incidence (panel A) and mortality (panel B), QALYs gained over net screening costs and cost-effectiveness acceptability (panel C, D), and life-years gained over required colonoscopies and burden-effectiveness acceptability (panel E, F). Bars represent means across model iterations, whiskers 95% uncertainty intervals (panel A, B); dots single iterations, solid circles means, dashed ellipses 95% uncertainty ranges (panel C, E); lines strategies' cost-effectiveness probabilities at a given cost or burden-acceptance threshold, squares strategies with the highest expected benefit (panel D, F). Strategies are denoted as "Test, Interval", where Colo denotes colonoscopy, FIT-X fecal immunochemical test with a cutoff equal to X, sDNA-FIT stool-DNA/FIT, LY life year, QALY quality-adjusted life year.

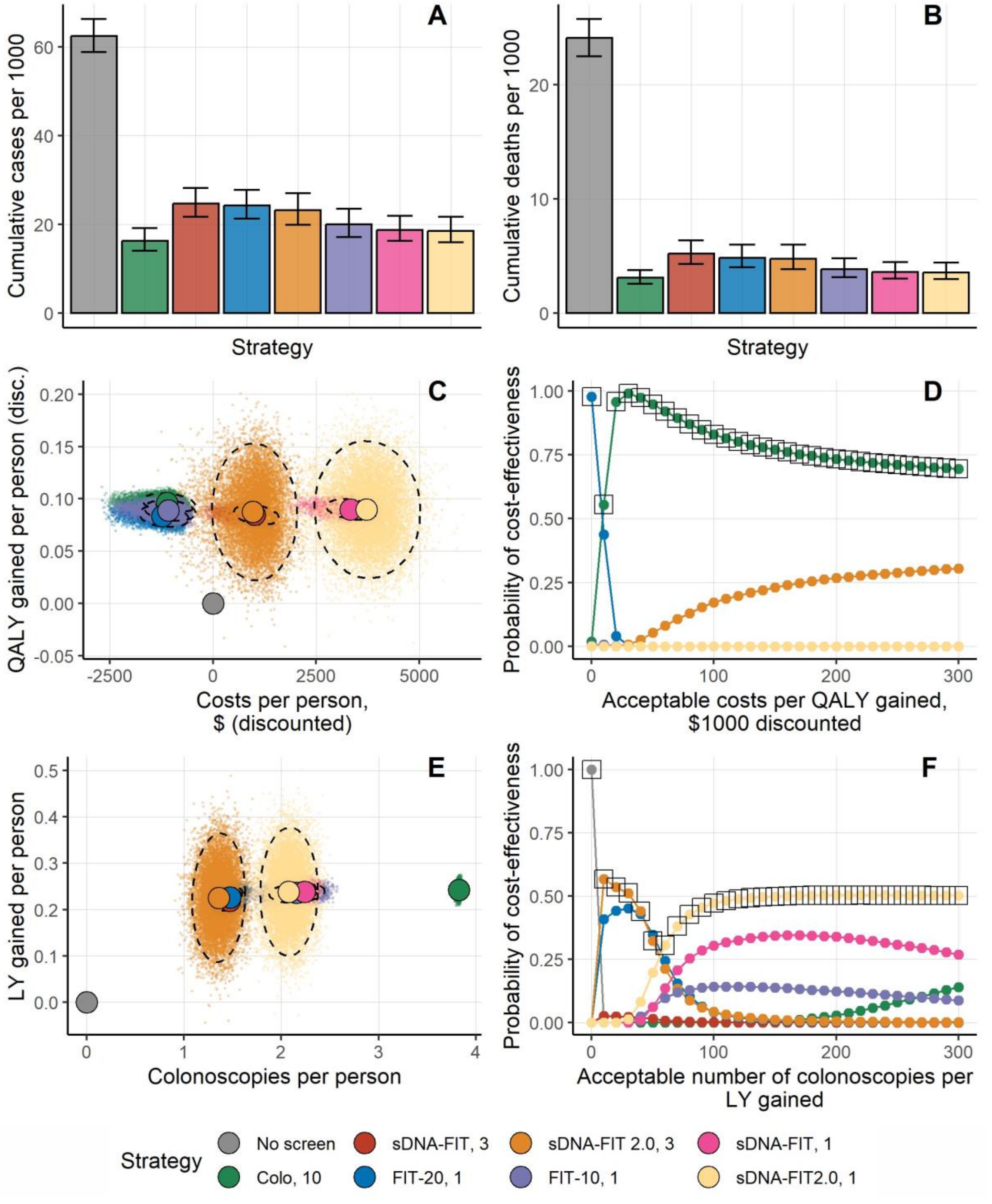

Supplementary Figure 8. Lifetime effectiveness, cost-effectiveness and burden-benefit of screening strategies: *high SSL prevalence* scenario.

In this scenario, we assumed two-fold increased prevalence and 50% reduced colonoscopy sensitivity for serrated lesions, based on empirical data suggesting a two-fold difference in detection between high-quality endoscopists relative to all endoscopists. In Panel F, cost-effectiveness refers to the ratio of additional colonoscopies per life-year gained.

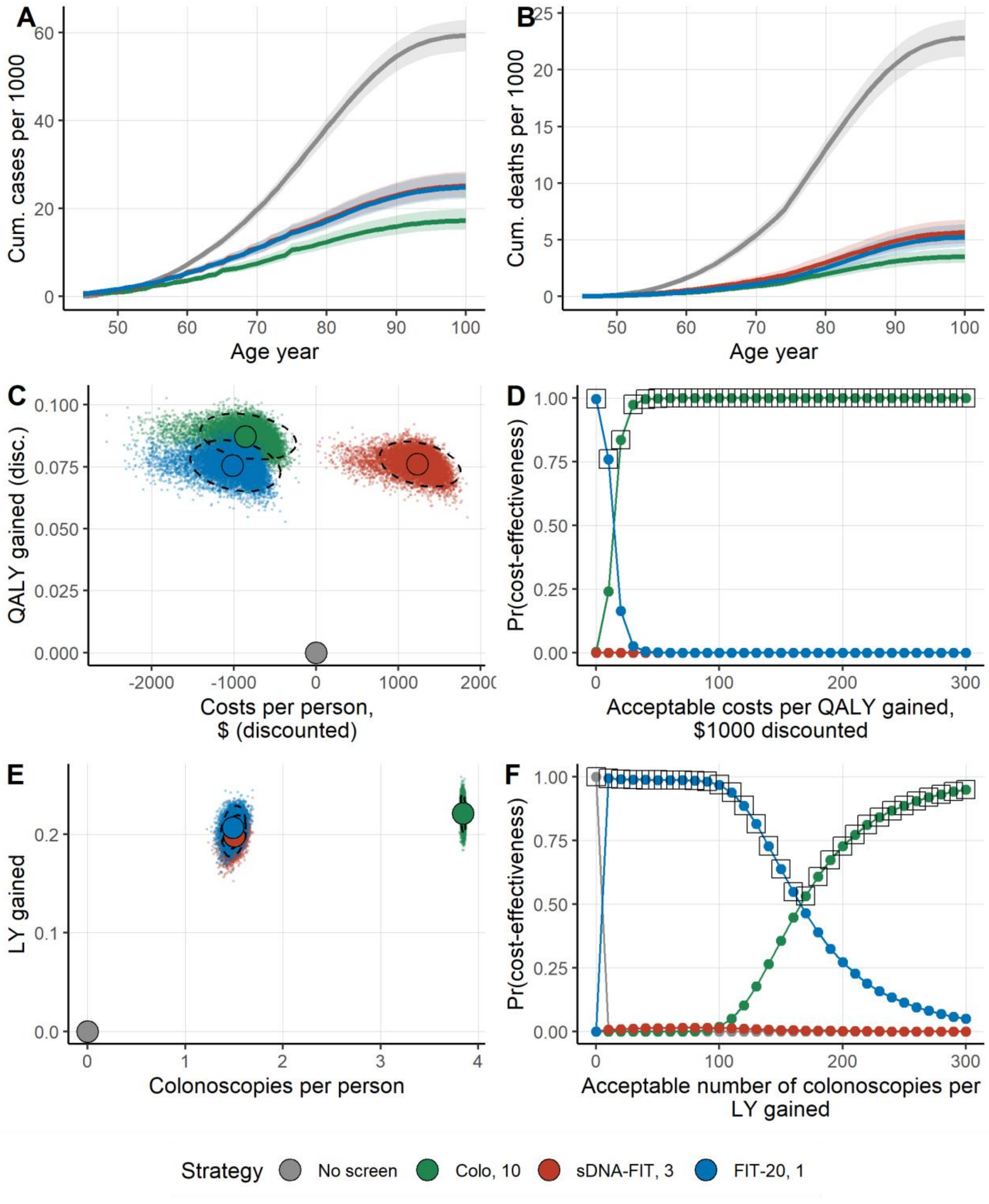

Supplementary Figure 9. Lifetime effectiveness, cost-effectiveness and burden-benefit of screening strategies: *high SSL risk* scenario.

In this scenario, we assumed two-fold increased progression rates for serrated lesions compared to adenomas, based on overrepresentation of serrated lesions among interval cancers. In Panel F, cost-effectiveness refers to the ratio of additional colonoscopies per life-year gained.

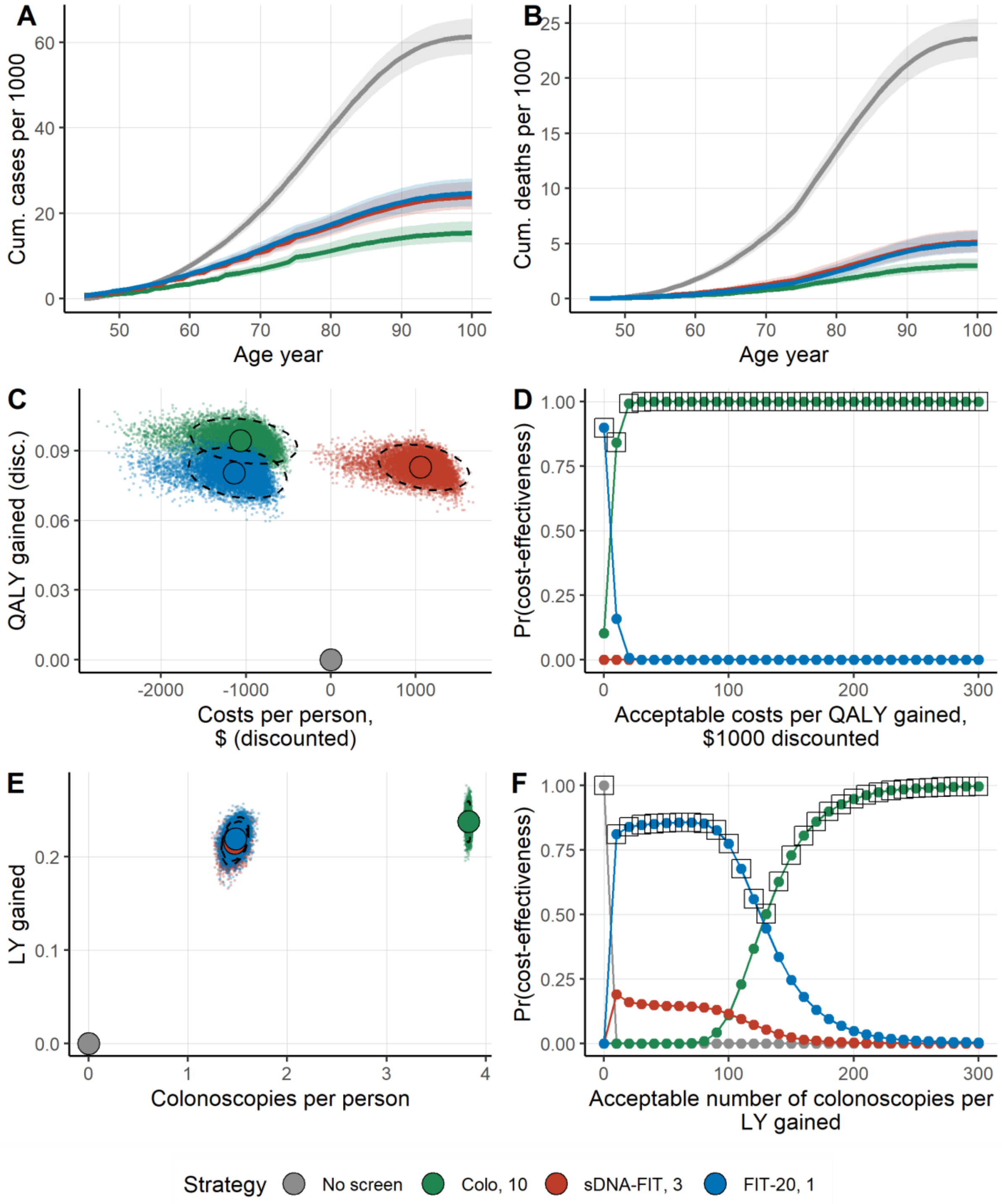

Supplementary Figure 10. Lifetime effectiveness, cost-effectiveness and burden-benefit of screening strategies: *high SSL prevalence and risk* scenario.

In this scenario, we assumed two-fold increased prevalence compared to the base-case and three-fold increased progression rates of serrated lesions compared to adenomas. In Panel F, cost-effectiveness refers to the ratio of additional colonoscopies per life-year gained.

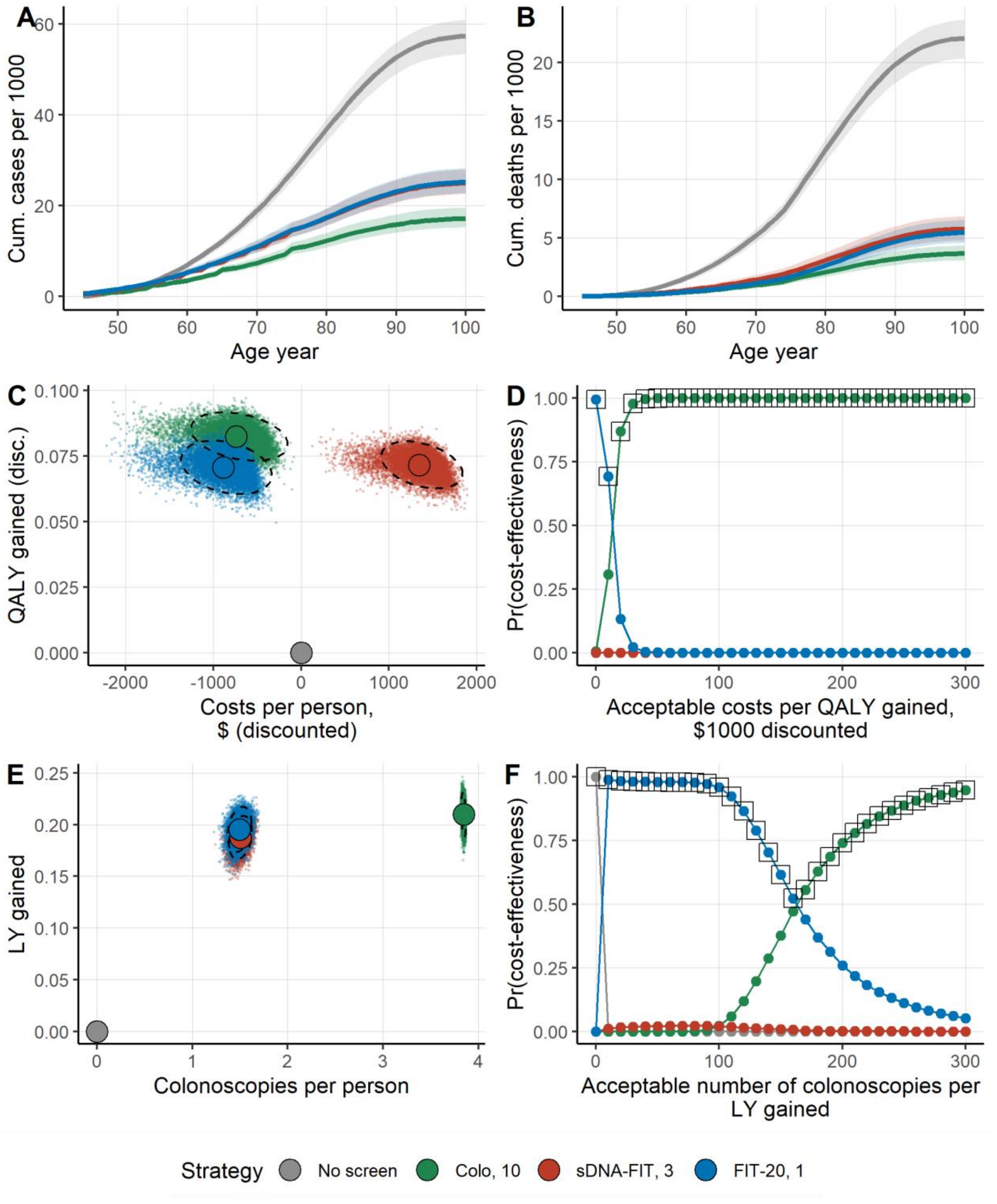

Supplementary Figure 11. Lifetime effectiveness, cost-effectiveness and burden-benefit of screening strategies: *increased background incidence reflecting early-onset trends*.

In this scenario, we assumed 1.54 increased incidence of CRC compared to the early 1990, consistent with trends in incidence <50 years. In Panel F, cost-effectiveness refers to the ratio of additional colonoscopies per life-year gained.

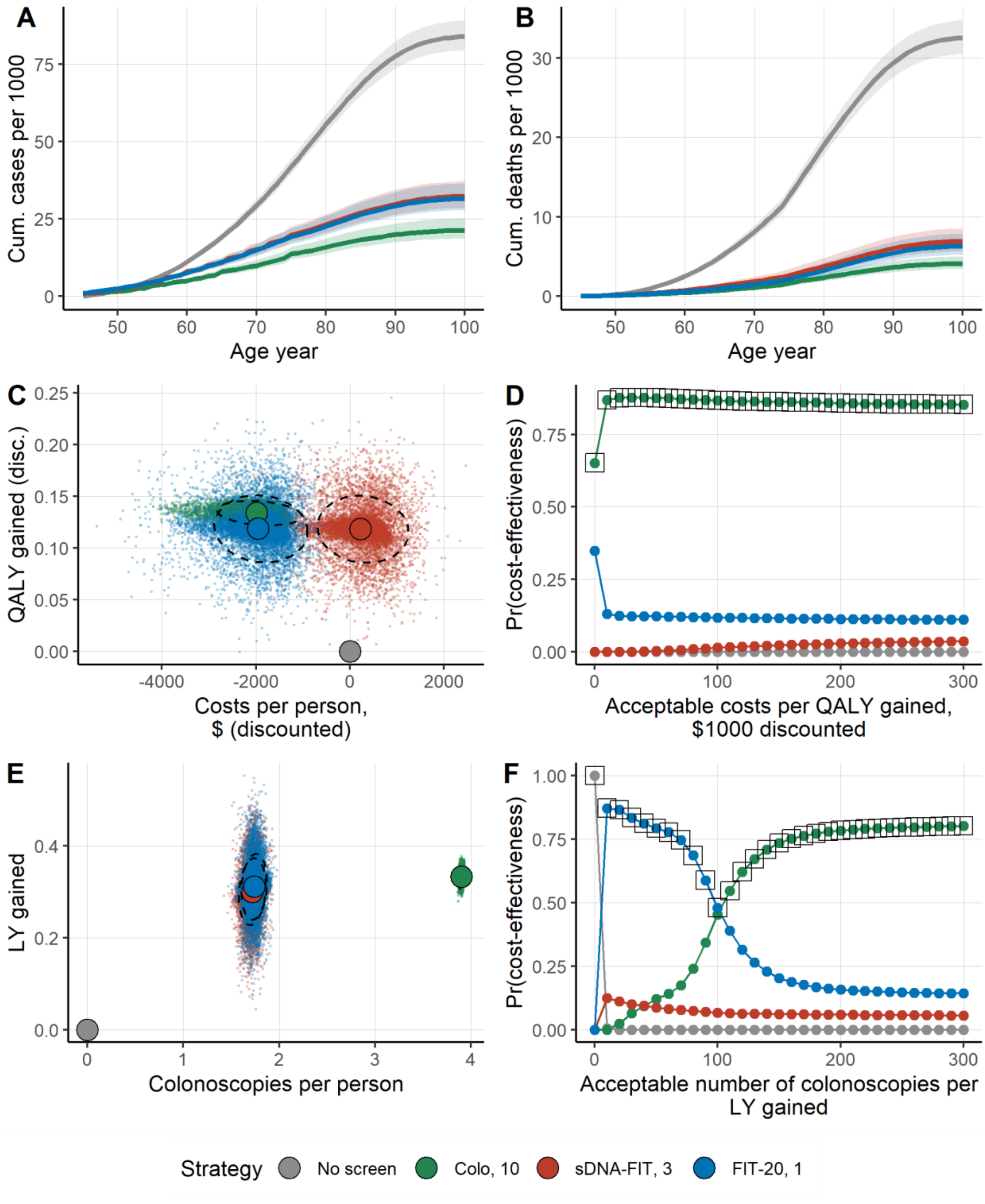

Supplementary Table 1. ANSER's natural history model form, parameters, and data sources.

Model component	Functional form	Parameters		Data targets	
	Time to event distribution	#	Type	#	Type
Initial adenoma formation	Weibull	3	Shape, scale defined by<br>sex	117	Prevalence by age and sex
Initial SSL formation	Weibull	3	,,	20	Prevalence by age, and by sex
Subsequent lesion formation	Exponential	8	Rate defined by<br>sex<br>number (2,3+)<br>type (adenoma, SSL)	6	Multiplicity, meta-estimate §
Lesion localization	Multinomial	6	Proportion proximal, distal, or rectal (1-former) by<br>type (adenoma, SSL)	6	Localization, meta-estimate
Lesion growth (time)	Size and lesion type-specific annual transition probability ‡	4	Probability defined by<br>size (small, large)<br>type (adenoma, SSL)	6	Size distribution, meta-estimate
Large lesion progression	Age and sex-specific annual transition probability ‡	8	Probability defined by<br>age (20-39, 40-59, …, 80+)<br>sex	112	CRC incidence by age and sex
Preclinical CRC stage progression	Age and stage-specific annual transition probability ‡	24	Probability defined by<br>age (20-39, 40-59, ..., 80+)<br>departure stage (AJCC I-IV)<br>destination ,, (+1-3 stages)	56	CRC incidence by age and stage
Preclinical CRC symptom development	Age and stage-specific annual transition probability ‡	16	Probability defined by<br>age (20-39, 40-59, ..., 80+)<br>preclinical stage (AJCC I-IV)	,,	,,
Post-removal lesion recurrence	Size and lesion type-specific annual transition probability ‡	6	Probability defined by<br>size (dim., small, large)<br>type (adenoma, SSL)	6	Detection of nonadvanced precursor lesions, advanced lesions and CRCs in surveillance
CRC survival	Empirical	n/a	-	-	CRC survival by age, sex, stage, location and time since diagnosis
Other-cause survival	Empirical	n/a	-	-	US lifetable by sex

* Weibull distribution; † Exponential distribution; ‡ With discontinuity correction (Simpson's 1/3rd rule); § the same multiplicity assumed by sex (4 parameters, 6 targets). AJCC denotes American Joint Committee on cancer staging; CRC colorectal cancer; SSL sessile serrated lesion; dim. diminutive.

Supplementary Table 2. ANSER parameters including 95%CIs for uncertainty analysis.

Model assumption	Mean	95% CI		Reference
Initial adenoma and SSL formation				Meester et al.[6]
Initial adenoma formation, shape *	1.36	1.18	1.63	
Initial adenoma formation, scale, F *	89.9	82.5	94.8	
Initial adenoma formation, scale, M *	75.9	69.6	82.4	
Initial SSL formation, shape *	1.56	1.38	1.75	
Initial SSL formation, scale, F *	311.0	270.3	347.7	
Initial SSL formation, scale, M	262.6	221.6	303.7	
Subsequent lesion formation				Meester et al.[6]
Second adenoma formation, annual rate, F	0.048	0.039	0.057	
Second adenoma formation, annual rate, M	0.057	0.045	0.068	
Further adenoma formation, annual rate, F	0.077	0.055	0.097	
Further adenoma formation, annual rate, M	0.092	0.068	0.109	
Second SSL formation, annual rate, F	0.023	0.017	0.028	
Second SSL formation, annual rate, M	0.027	0.020	0.034	
Further SSL formation, annual rate, F	0.054	0.031	0.078	
Further SSL formation, annual rate, M	0.064	0.036	0.098	
Precursor growth				Meester et al.[6]
Adenoma annual growth rate, diminutive>small ‡	0.063	0.055	0.071	
Adenoma annual growth rate, small>large ‡	0.025	0.021	0.030	
SSL annual growth rate, diminutive>small ‡	0.057	0.046	0.069	
SSL annual growth rate, small>large ‡	0.042	0.029	0.058	
Precursor localization				Meester et al.[6]
Adenoma localization, proximal †	0.593	-	-	
Adenoma localization, distal †	0.342	-	-	
Adenoma localization, rectal †	0.062	-	-	
SSL localization, proximal †	0.715	-	-	
SSL localization, distal †	0.204	-	-	
SSL localization, rectal †	0.074	-	-	
Precursor progression				SEER 1990-94
Annual progression rate to CRC, ages 20-39y, F	0.014	0.010	0.017	
Annual progression rate to CRC, ages 40-59y, F	0.016	0.012	0.022	
Annual progression rate to CRC, ages 60-79y, F	0.018	0.014	0.023	

Annual progression rate to CRC, ages 80-99y, F	0.019	0.015	0.024	
Annual progression rate to CRC, ages 20-39y, M	0.015	0.011	0.019	
Annual progression rate to CRC, ages 40-59y, M	0.017	0.012	0.023	
Annual progression rate to CRC, ages 60-79y, M	0.019	0.015	0.025	
Annual progression rate to CRC, ages 80-99y, M	0.020	0.016	0.026	
<u>CRC stage progression by age</u>				SEER 1990-94
Annual progression rate, stage I>II, ages 20-39y	0.563	0.521	0.622	
Annual progression rate, stage I>II, ages 40-59y	0.309	0.290	0.313	
Annual progression rate, stage I>II, ages 60-79y	0.281	0.261	0.286	
Annual progression rate, stage I>II, ages 80-99y	0.330	0.279	0.348	
Annual progression rate, stage I>III, ages 20-39y	0.161	0.049	0.223	
Annual progression rate, stage I>III, ages 40-59y	0.064	0.000	0.114	
Annual progression rate, stage I>III, ages 60-79y	0.056	0.002	0.095	
Annual progression rate, stage I>III, ages 80-99y	0.051	0.000	0.095	
Annual progression rate, stage I>IV, ages 20-39y	0.028	0.000	0.080	
Annual progression rate, stage I>IV, ages 40-59y	0.039	0.000	0.078	
Annual progression rate, stage I>IV, ages 60-79y	0.032	0.000	0.067	
Annual progression rate, stage I>IV, ages 80-99y	0.025	0.000	0.060	
Annual progression rate, stage II>III, ages 20-39y	0.317	0.280	0.326	
Annual progression rate, stage II>III, ages 40-59y	0.346	0.331	0.367	
Annual progression rate, stage II>III, ages 60-79y	0.294	0.273	0.327	
Annual progression rate, stage II>III, ages 80-99y	0.258	0.233	0.293	
Annual progression rate, stage II>IV, ages 20-39y	0.020	0.000	0.035	
Annual progression rate, stage II>IV, ages 40-59y	0.037	0.000	0.076	
Annual progression rate, stage II>IV, ages 60-79y	0.041	0.000	0.083	
Annual progression rate, stage II>IV, ages 80-99y	0.041	0.000	0.073	
Annual progression rate, stage III>IV, ages 20-39y	0.251	0.216	0.277	
Annual progression rate, stage III>IV, ages 40-59y	0.240	0.219	0.260	
Annual progression rate, stage III>IV, ages 60-79y	0.235	0.213	0.252	
Annual progression rate, stage III>IV, ages 80-99y	0.256	0.243	0.265	
<u>CRC symptom presentation by age</u>				SEER 1990-94
Annual presentation rate in stage I, ages 20-39y	0.100	0.087	0.111	
Annual presentation rate in stage I, ages 40-59y	0.103	0.071	0.126	
Annual presentation rate in stage I, ages 60-79y	0.104	0.072	0.128	
Annual presentation rate in stage I, ages 80-99y	0.091	0.061	0.113	
Annual presentation rate in stage II, ages 20-39y	0.210	0.118	0.286	
Annual presentation rate in stage II, ages 40-59y	0.311	0.219	0.395	
Annual presentation rate in stage II, ages 60-79y	0.387	0.279	0.489	
Annual presentation rate in stage II, ages 80-99y	0.405	0.315	0.496	

Annual presentation rate in stage III, ages 20-39y	0.416	0.343	0.497	
Annual presentation rate in stage III, ages 40-59y	0.589	0.556	0.629	
Annual presentation rate in stage III, ages 60-79y	0.607	0.574	0.654	
Annual presentation rate in stage III, ages 80-99y	0.585	0.566	0.613	
Annual presentation rate in stage IV, ages 20-39y	0.789	0.759	0.821	
Annual presentation rate in stage IV, ages 40-59y	0.887	0.875	0.902	
Annual presentation rate in stage IV, ages 60-79y	0.916	0.901	0.935	
Annual presentation rate in stage IV, ages 80-99y	0.903	0.893	0.915	
Colorectal cancer survival				SEER 2004-15
CRC survival rate by age, sex, stage, location and time since diagnosis §	Empirical			
Colonoscopy complications				Lin et al.[30]
Colonoscopy-related bleeding rate	0.00146	0.00094	0.00199	
Colonoscopy-related bowel perforation rate	0.00031	0.00023	0.00039	
Cost assumptions ‖				CMS
Fecal immunochemical test (FIT)	18.0	17.5	19.0	
Stool DNA-FIT (sDNA-FIT)	509	495	536	
Colonoscopy without polypectomy	741	677	871	
Colonoscopy with polypectomy	1,055	963	1,239	
Colonoscopy-related bleeding	6,369	5,202	9,334	
Colonoscopy-related perforation	17,429	14,175	25,434	
Stage I CRC, treatment year 1	43,321	30,068	54,211	Mariotto et al.[36]
Stage I CRC, treatment years 2-5	4,456	3,086	5,564	
Stage I CRC, terminal year (superseding above)	87,624	60,677	109,398	
Stage II CRC, treatment year 1	61,578	42,642	76,880	
Stage II CRC, treatment years 2-5	5,196	3,598	6,488	
Stage II CRC, terminal year (superseding above)	98,778	68,401	123,324	
Stage III CRC, treatment year 1	89,608	62,003	110,536	
Stage III CRC, treatment years 2-5	8,064	5,580	9,947	
Stage III CRC, terminal year (superseding above)	103,592	71,679	127,787	
Stage IV CRC, treatment year 1	133,160	92,139	164,261	
Stage IV CRC, treatment years 2-5	31,877	26,416	47,094	
Stage IV CRC, terminal year (superseding above)	130,259	90,131	160,683	
Disutility assumptions				
Stool tests	0.00055	0.00031	0.00079	Expert opinion
Colonoscopy	0.00180	0.00095	0.00265	
Colonoscopy-related bleeding	0.00555	0.00294	0.00816	

Colonoscopy-related perforation	0.01920	0.01008	0.02833	
Stage I CRC, treatment year 1	0.12	0.07	0.18	Ness et al.[39]
Stage I CRC, treatment years 2-5	0.06	0.04	0.09	
Stage I CRC, terminal year (superseding above)	0.12	0.07	0.18	
Stage II CRC, treatment year 1	0.19	0.13	0.25	
Stage II CRC, treatment years 2-5	0.09	0.06	0.13	
Stage II CRC, terminal year (superseding above)	0.19	0.13	0.25	
Stage III CRC, treatment year 1	0.24	0.18	0.31	
Stage III CRC, treatment years 2-5	0.12	0.09	0.15	
Stage III CRC, terminal year (superseding above)	0.24	0.18	0.31	
Stage IV CRC, treatment year 1	0.69	0.63	0.76	
Stage IV CRC, treatment years 2-5	0.35	0.32	0.38	
Stage IV CRC, terminal year (superseding above)	0.69	0.63	0.76	

Diagnostic test characteristics

See **Table 1** from the article.

CRC denotes colorectal cancer; F female; M male; SSL sessile serrated lesion.

* The time to initial lesion formation followed a Weibull distribution with specified shape and scale parameters.

† Location was categorized as proximal (cecum-splenic flexure), distal (splenic flexure-rectosigmoid colon), rectal (rectum). Lesion localization variation was replicated using fixed parameters (Supplementary Figure 3), so these assumptions were not varied.

‡ Size of lesions was categorized as diminutive (diameter of 0-5 mm), small (6-9 mm), large (≥10mm).

§ See Supplementary Figure 5 for a visual summary of survival by age, sex, stage, location and time since diagnosis.

ǁ See Supplementary Figure 6 for the empiric basis behind the assumed cost variation.

Supplementary Table 3. Cross-model comparison in outcomes.

			Model outcomes per 1000 adults (95% uncertainty intervals)					
**Strategy**	**IRR***	**Model**	Cases	Deaths	LYG	Cost, $ thousands	Stool tests	Exams
No screening	1	ANSER	63 (59-66)	24 (22-26)	-	3,200 (2,600-4,400)	-	63 (59-66)
	1	SimCRC[52]	70	28	-	n/a	-	70
	1	MISCAN[38,52]	67	28	-	4,560 †	-	67
	1	Ladabaum[25]	59	23	-	3020	-	59
	1.54	ANSER	84 (79-89)	32 (30-35)	-	4,300 (3,500-6,000)	-	84 (79-89)
	1.19	SimCRC[53]	85	34	-	n/a	-	85
	1.19	MISCAN[53]	81	34	-	n/a	-	81
Colo 45-75, 10	1	ANSER	16 (14-19)	3 (3-4)	242 (223-262)	2,000 (1,800-2,500)	-	3,888 (3,861-3,912)
	1	SimCRC[52]	9	2	303	n/a	-	4853
	1	MISCAN[38,52]	23	5	262	6000 †	-	4928
	1	Ladabaum[25]	16	5	n/a	4170	-	n/a
	1.54	ANSER	21 (19-25)	4 (3-5)	333 (309-357)	2,300 (2,000-2,800)	-	3,986 (3,962-4,013)
	1.19	SimCRC[53]	14	3	369	n/a	-	4212
	1.19	MISCAN[53]	34	8	301	n/a	-	4232
FIT 45-75, 1	1	ANSER	24 (21-28)	5 (4-6)	226 (205-246)	1,900 (1,700-2,400)	18,735 (17,891-19,558)	1,536 (1,415-1,658)
	1	SimCRC[52]	20	4	311	n/a	19,196	1,979
	1	MISCAN[38,52]	34	7	247	4,760 †	19,256	1,995
	1	Ladabaum[25]	23	5	n/a	2,400	n/a	n/a
	1.54	ANSER	34 (28-36)	6 (5-8)	311 (212-411)	2,400 (2,000-3,000)	17,531 (16,691-18,368)	1,823 (1,700-1,944)
	1.19	SimCRC[53]	26	6	348	n/a	19,680	1,602

1.19	MISCAN[53]	46	10	291	n/a	19,607	1,620

Numbers represent model best-estimates, in ANSERs case, from best-fitting natural history parameters. Strategies are denoted as "Test, Interval." Colo denotes colonoscopy; FIT-X fecal immunochemical test with cutoff X microgram Hb per gram stool; IRR incidence rate ratio; LYG life-year gained.

* Assumed incidence rate ratio compared to the models' original source data, to account for trends in early-onset cancer.

† Costs are from a different study than other outcomes, the cited paper by Peterse et al.

Supplementary Table 4. Lifetime effectiveness, burden and cost for all evaluated strategies.

**Outcomes**	**Strategy**						
Mean (95% UI)	Colonoscopy every 10 yrs	sDNA-FIT every 3 yrs	FIT-20 every 1 yr	sDNA-FIT 2.0 every 3 yrs	FIT-10 every 1 yr	sDNA-FIT every 1 yr	sDNA-FIT 2.0 every 1 yr
CRC cases averted per 1000 persons	46.3 (42.6-50)	37.9 (34.1-41.6)	38.3 (34.4-42)	39.4 (34.5-44.3)	42.5 (38.5-46.5)	43.8 (40-47.5)	44.1 (39.7-48.5)
% reduction vs no screening	74.0% (69.6-77.3%)	60.6% (55.5-64.9%)	61.2% (56.1-65.6%)	62.9% (56.5-68.4%)	68.0% (62.8-72.3%)	70.0% (65.2-73.6%)	70.5% (65.3-74.6%)
CRC deaths averted per 1000 persons	21.0 (19.5-22.5)	18.9 (17.3-20.5)	19.3 (17.6-20.8)	19.3 (17.4-21.2)	20.3 (18.7-21.8)	20.5 (18.9-22.1)	20.5 (18.8-22.3)
% reduction vs no screening	87.3% (84.5-89.2%)	78.5% (73.8-82%)	80.0% (75.4-83.1%)	80.3% (75.0-84.2%)	84.2% (80.3-86.8%)	85.2% (81.7-87.5%)	85.3% (81.6-87.8%)
Life-years gained per 1000 persons, undiscounted	242 (223-262)	219 (198-239)	226 (205-246)	225 (100-352)	236 (216-256)	238 (218-258)	239 (114-366)
QALYs gained per person, discounted *	0.096 (0.088-0.105)	0.085 (0.076-0.093)	0.083 (0.074-0.093)	0.088 (0.028-0.148)	0.089 (0.08-0.098)	0.09 (0.081-0.099)	0.09 (0.03-0.15)
Incremental cost per person, discounted $ *	-1,100 (-2000 to 700)	1,000 (300-1,400)	-1,200 (-2,000 to 800)	1,000 (-300 to 1,800)	-1,100 (-1,900 to 700)	3,300 (2,600-3,800)	3,700 (2,400-4,800)
Cost per QALY gained vs no screening, discounted $	Colonoscopy dominates	12,100 (3,300-17,700)	FIT-20 dominates	13,700 (sDNA-FIT2.0 dominates – 40,900)	FIT-10 dominates	37,200 (27,800-45,200)	48,600 (20,900-125,500)
Cost per QALY gained vs FIT-20, discounted $ †	9,300 (500-21,900)	792,400 (FIT-20 dominates –12.8 million)	-	40,500 (FIT-20 dominates – 1.2 million)	26,200 (4,800-83,700)	697,800 (446,200-1.4 million)	100,800 (FIT-20 dominates – 2.4 million)

Number of stool tests per person	-	7.0 (6.8-7.3)	18.7 (17.9-19.6)	7.3 (6.8-7.9)	13.2 (11.3-15.4)	12.6 (11.9-13.4)	14.1 (12.0-16.4)
Number of colonoscopies per person	3.9 (3.8-3.9)	1.5 (1.4-1.6)	1.5 (1.4-1.6)	1.4 (1.1-1.6)	2.2 (1.9-2.4)	2.2 (2.1-2.4)	2.1 (1.8-2.3)

CRC denotes colorectal cancer; FIT-X fecal immunochemical test with cutoff X; sDNA-FIT stool-DNA/FIT; QALY quality-adjusted life year; UI uncertainty interval.

* Marked outcomes are discounted by 3% per year.

† Medians are reported for costs per QALY gained vs. FIT-20 instead of means, which were strongly influenced by outliers.

Supplementary Table 5. Optimal among all evaluated strategies in sensitivity analyses.

Outcome (95%UI)	Scenario				
	Base case	Increased SSL prevalence	Increased SSL progression	Increased SSL prevalence & progression	Increased overall CRC incidence
Fraction of CRC arising from SSLs, %	11.8 (8.1-15.8)	16.6 (13.0-20.3)	20.1 (14.6-25.5)	23.1 (18.8-26.8)	-
Maximum CRC mortality reduction, %					
Colonoscopy, 10	86.4	89.7	88.3	88.7	84.4
sDNA-FIT, 3	0	0	0	0	0.0
FIT-20, 1	0	0	0	0	0
sDNA-FIT2.0, 3	0	0	0	0.0	0
FIT-10, 1	0	0.0	0	0.0	0.7
sDNA-FIT, 1	0.2	0.1	0.2	0.1	6.3
sDNA-FIT2.0, 1	13.4	10.1	11.5	9.0	8.6
Maximum QALY s.t. $100k/QALY gained, %					
Colonoscopy, 10	82.9	83.2	83.2	81.6	56.8
sDNA-FIT, 3	0	0	0	0	0.8
FIT-20, 1	0	0	0	0	7.9
sDNA-FIT2.0, 3	17.1	16.8	16.8	16.7	0
FIT-10, 1	0	0	0	0.0	34.4
sDNA-FIT, 1	0	0	0	0	0
sDNA-FIT2.0, 1	0	0	0	1.7	0
Maximum life-years s.t. 100 colonoscopies/LY gained, %					
Colonoscopy, 10	0	0	0	0	22.8
sDNA-FIT, 3	0.1	0.0	0.1	0.1	4.5
FIT-20, 1	4.1	11.2	1.6	9.4	24.3
sDNA-FIT2.0, 3	4.2	4.8	4.0	24.0	0.2
FIT-10, 1	14.1	18.6	10.2	13.8	4.0
sDNA-FIT, 1	30.4	19.5	36.7	15.0	11.2
sDNA-FIT2.0, 1	47.2	45.8	47.3	37.8	33.0
Cost per QALY gained, sDNA-FIT, 3 vs FIT-20, $	792,400 (FIT-20 dominates – 12.9 million)	722,700 (FIT-20 dominates – 17.1 million)	701,600 (FIT-20 dominates – 8.9 million)	750,000 (FIT-20 dominates – 15.6 million)	FIT-20 dominates (FIT-20 dominates – 10.7 million)
Cost per QALY gained, sDNA-FIT 2.0, 3 vs FIT-10, $	FIT-10 dominates (FIT-10 dominates – 1.1 million)	FIT-10 dominates (FIT-10 dominates – 1.1 million)	FIT-10 dominates (FIT-10 dominates – 1.1 million)	FIT-10 dominates (FIT-10 dominates – 990,700)	FIT-10 dominates (FIT-10 dominates – 2.8 million)

Strategies are denoted as “Test, interval”. CRC denotes colorectal cancer; FIT-X = fecal immunochemical test with cutoff X; sDNA-FIT stool-DNA/FIT; QALY quality-adjusted life year; SSL sessile serrated lesion; s.t. subject to; UI uncertainty interval.
